# AoSte12 Is Required for Mycelial Development, Conidiation, Trap Morphogenesis, and Secondary Metabolism by Regulating Hyphal Fusion in Nematode-Trapping Fungus *Arthrobotrys oligospora*

**DOI:** 10.1128/spectrum.03957-22

**Published:** 2023-02-14

**Authors:** Na Bai, Meihua Xie, Qianqian Liu, Wenjie Wang, Yankun Liu, Jinkui Yang

**Affiliations:** a State Key Laboratory for Conservation and Utilization of Bio-Resources and Key Laboratory for Microbial Resources of the Ministry of Education, School of Life Sciences, Yunnan University, Kunming, People’s Republic of China; b School of Life Sciences, Yunnan University, Kunming, People’s Republic of China; Broad Institute

**Keywords:** transcription factor Ste12, hyphal fusion, trap formation, conidiation, secondary metabolism, stress response

## Abstract

Nematode-trapping (NT) fungi are a unique group of carnivorous microorganisms that can capture and digest nematodes by producing ingenious trapping devices (traps). *Arthrobotrys oligospora*, a representative NT fungus, can develop adhesive three-dimensional networks for nematode predation. Hyphal fusion is indispensable for the trap formation of *A. oligospora*. Here, we characterized an orthologous Ste12 protein (AoSte12) in *A. oligospora* via gene disruption, DNA affinity purification sequencing (DAP-Seq), and multi-omics approaches. The disruption of the *Aoste12* gene caused an increase in hyphal fusion and resulted in defects in mycelial growth, conidiation, trap morphology, and stress resistance, as well as reducing the number of nuclei and lipid droplet accumulation. Moreover, transcriptome and DAP-Seq analysis revealed that AoSte12 was involved in cellular processes associated with growth, cell fusion, the tricarboxylic acid cycle, vesicles, actin filaments, and lipid metabolism. In addition, combining metabolome with transcriptome and DAP-Seq analysis indicated that AoSte12 was involved in the mitogen-activated protein kinase signaling pathway, lipid metabolism, and secondary metabolites. A yeast two-hybrid assay revealed that AoSte12 can interact with diverse proteins, such as the MAK-2 orthologue protein Fus3, the vacuolar sorting protein Pep3, and UDP-glycosyltransferase. Our results suggest that AoSte12 plays an indispensable role in hyphal fusion and thus regulates sporulation and trap morphogenesis. These results provide deep insights into the connection between hyphal fusion and trap formation in NT fungi.

**IMPORTANCE** Nematode-trapping (NT) fungi are an important natural enemy of nematodes and can capture their prey by producing traps. Hyphal anastomosis and fusion are important for mycelial growth and the colony morphological development of filamentous fungi and are also crucial for the trap morphogenesis of NT fungi. *Arthrobotrys oligospora* can form complex three-dimensional networks (traps) when sensing the presence of nematodes. This study revealed that AoSte12 is indispensable for hyphal fusion and that it regulates mycelial growth, conidiation, trap morphogenesis, stress resistance, the number of nuclei, and lipid droplet accumulation in *A. oligospora*. In addition, DNA affinity purification sequencing, transcriptome, and metabolome analyses further revealed that AoSte12 is involved in the mitogen-activated protein kinase pathway, lipid metabolism, and secondary metabolism. Overall, these findings expand the important role of AoSte12 in NT fungus *A. oligospora* and provide a broad foundation for elucidating the regulatory mechanism of trap development and the lifestyle transitions of pathogenic fungi.

## INTRODUCTION

Nematode-trapping (NT) fungi are a special ecological group that can use their own specialized structures (traps) to capture nematodes that move freely in the soil. Most NT fungi can live both saprophytically on organic matter and, as predators, by capturing tiny animals (1–3). NT fungi change their saprophytic lifestyle to a predacious lifestyle by producing traps, so traps are not only the weapons that NT fungi use to attack nematodes but are also an important indicator of their lifestyle transition (4, 5). As potential biocontrol agents, the origin of NT fungi and the regulation mechanism of trap formation and secondary metabolites have attracted extensive interest (6, 7). Arthrobotrys oligospora is a typical NT fungus that produces an adhesive three-dimensional network, and it is often used as a potential model for studying the interactions between fungi and nematodes (8, 9). In recent years, multiple signaling pathways and cellular processes, including the mitogen-activated protein kinase (MAPK) (7, 10), G protein signaling (11–13), small GTPases (14, 15), autophagy (2, 16), and peroxisome (17) have been shown to involve in mycelial development and trap formation of NT fungus in *A. oligospora*.

The formation of traps in *A. oligospora* initially involves a ring structure; on this basis, multiple ring structures are formed, and they are finally combined to form a three-dimensional network; the combination of these ring structures is closely related to the fusion between the hyphae (18). Hyphal fusion is manifested in conidial anastomosis tubes formed between conidia; it is also reflected in the stage of hyphal growth and development (19). Moreover, in the pathogenic stage of NT fungi, the initial stages of trap formation also require hyphal fusion (18). Similar to cell fusion in other organisms, the process of hyphal fusion requires cell recognition, adhesion, and membrane merging (20). Hyphal fusion is regulated by many aspects, including MAPK cascades, reactive-oxygen-species (ROS)-generating systems, cell polarity factors, Ca^2+^ signaling factors, and the so-called STRIPAK (striatin interacting phosphatase and kinase) complex (19). The MAK-1/ADV-1 pathway and the MAK-2/PP-1 pathway engage in cross talk, and both pathways form a regulatory network that mediates growth, communication, and fusion (20). Recently, an orthologue of the Neurospora crassa hyphal anastomosis gene *sofT*, which is involved in hyphal anastomosis and cell-to-cell communication, was identified in the NT fungus Arthrobotrys flagrans (Duddingtonia flagrans); the absence of *sofT* caused a reduction in aerial mycelia, incomplete trap closure, and the absence of hyphal anastomoses (21). Therefore, hyphal fusion plays an important role in the formation of three-dimensional networks of *Arthrobotrys* species.

Ste12 is a C2H2 zinc finger protein transcription factor, which is homologous with the pheromone response pathway transcription factor Ste12 in Saccharomyces cerevisiae (22). Ste12 transcription factors, including PP-1, contain two C2H2 zinc finger protein motifs and an STE domain involved in binding DNA. In N. crassa, the STE domain is crucial for the function of transcription factors (23). Ste12 transcription factors are necessary for the cell fusion of Neurospora crassa and Trichoderma atroviride (24); however, for Aspergillus oryzae (25), Fusarium oxysporum (26), and the plant endophytic fungus Epichloë festucae (27), the absence of the *ste12* gene has no effect on cell fusion. Ste12 transcription factors are also involved in the regulation of fungal growth, virulence, dimorphic morphological transformation, and sexual and asexual development (28). For example, Ste12 regulates the dimorphic morphological transformation of yeast cells in response to starvation (29). In plant- and animal-pathogenic fungi such as Magnaporthe oryzae (30), Metarhizium acridum (31), and Fusarium oxysporum (26), Ste12 plays an important regulatory role in appressorium formation and pathogenicity. Recently, an orthologous Ste12 was identified in *A. oligospora* (strain TWF154); the disruption of *ste12* resulted in a defect in the formation of aerial hyphae and traps (32). However, the function and regulatory mechanism of Ste12 in the growth and development of *A. oligospora* and other NT fungi remain largely unknown.

In this study, we investigated the homologous protein Ste12 (AoSte12) in *A. oligospora* via multiphenotypic comparison. AoSte12 plays a crucial role in hyphal fusion and is involved in conidiation, trap morphogenesis, and lipid droplet (LD) accumulation. We also carried out transcriptome, DNA affinity purification sequencing (DAP-Seq), and yeast two-hybrid (Y2H) analyses to further excavate the downstream target genes of *Aoste12*. In addition, metabolome analysis was carried out for probing the role of AoSte12 in secondary metabolism.

## RESULTS

### Analysis of protein sequence and conserved domains of AoSte12.

The homologous protein AoSte12 (AOL_s00079g294) in *A. oligospora* was obtained by comparing the amino acid sequence of N. crassa Ste12 (XP_957811). AoSte12 contains 686 amino acids, and the predicted molecular weight and isoelectric point were 77 kDa and 6.33, respectively. The orthologs of Ste12 in the NT fungi and other filamentous fungi were downloaded and analyzed through BLAST comparison. The orthologs of Ste12 from NT fungi form an independent evolutionary branch (see Fig. S1A in the supplemental material). All Ste12 homologous proteins contain the Ste12 domain (IPR003120), and they also contain two C2H2 domains (IPR013087), except for Purpureocillium lilacinum and S. cerevisiae (see Fig. S1B). These orthologs of Ste12 in different fungi share a high degree of sequence similarity, among which the sequence similarity between AoSte12 and homologous proteins from five NT fungi is 89.2 to 96.4%, and the sequence similarity between AoSte12 and homologous proteins from other filamentous fungi is 51.3 to 54.8% (see Fig. S1C).

### Disruption of *Aoste12*.

The gene *Aoste12* was disrupted using the homologous recombination method (see Fig. S2A); the positive transformants were screened on PDAS medium (potato dextrose agar [PDA] supplemented with 0.6 M sucrose) containing 200 μg/mL hygromycin and verified via PCR with the primers Ste12F and Ste12R (see Fig. S2B and Table S1 in the supplemental material). Southern blotting was used for further verification. The genomic DNA of the wild-type (WT) and mutant strains was digested using the restriction enzyme *MfeI*, and a North2South chemiluminescence hybridization and detection kit was used to verify the WT and mutant strains (see Fig. S2C). Finally, three independent transformants (Δ*Aoste12-8*, Δ*Aoste12-9*, and Δ*Aoste12-13*) were confirmed, and one of them (Δ*Aoste12-8*) was selected for subsequent analysis due to their phenotypes being consistent.

### AoSte12 regulates mycelial growth and stress resistance.

**FIG 1:**
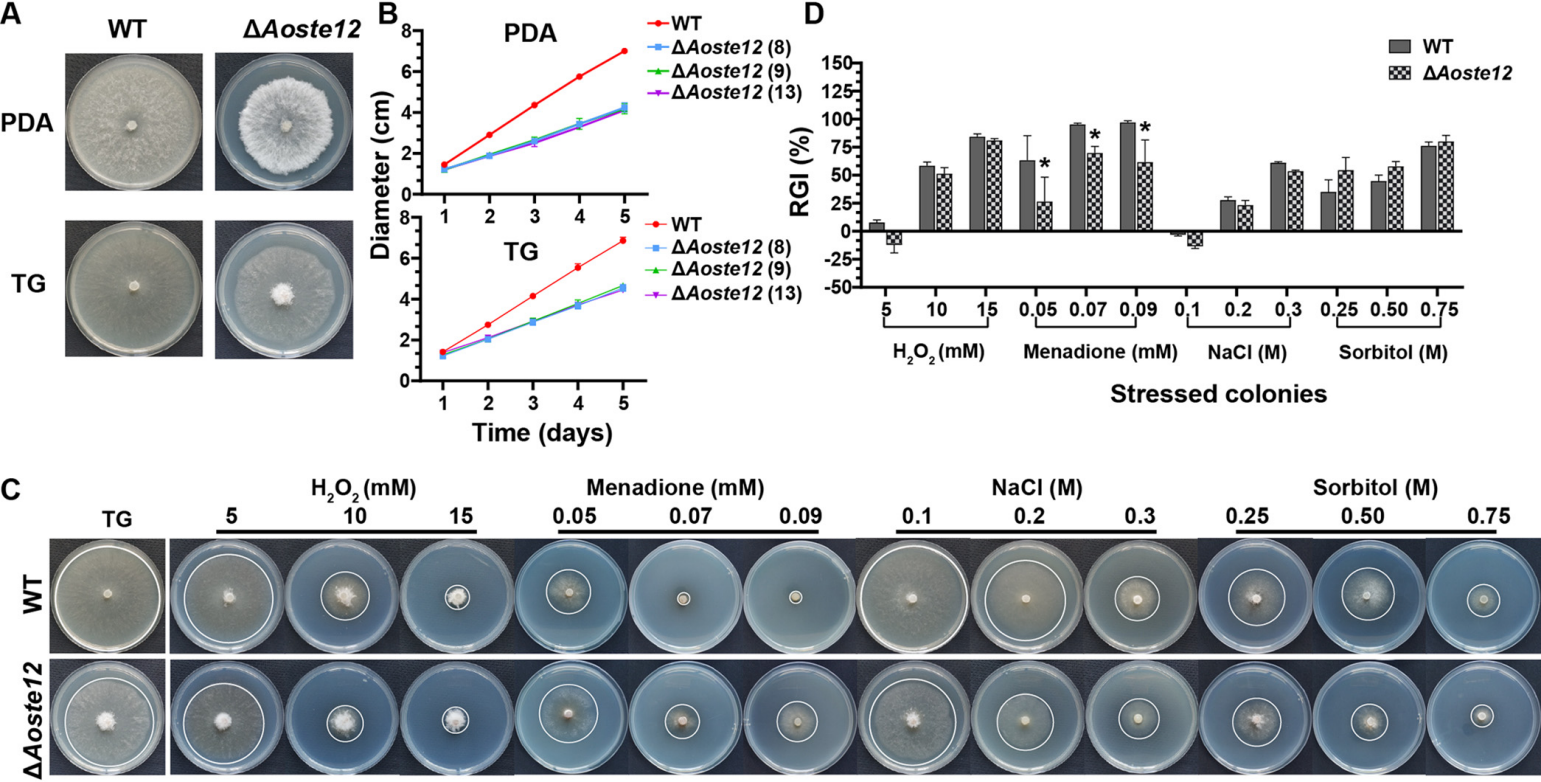
Comparison of mycelial growth and stress tolerance between wild-type (WT) and Δ*Aoste12* mutant strains. (A) Colonial morphologies of WT and Δ*Aoste12* mutant incubated on PDA and TG medium at 28°C for 5 days. (B) Mycelial growth rate of WT and Δ*Aoste12* mutants incubated on PDA and TG media. The numbers in parentheses after the strain names indicate the three independent Δ*Aoste12* mutants. (C) Colonial morphologies of WT and Δ*Aoste12* mutant in TG medium and medium supplemented with chemical agents (H_2_O_2_, menadione, NaCl, and sorbitol). The edge of colony was marked with a white circle. (D) Relative growth inhibition (RGI) of WT and Δ*Aoste12* mutant in the medium described above. Error bars represent the standard deviations. An asterisk indicates a significant difference between the mutant and the WT strain (Tukey’s HSD, *P < *0.05).

WT and mutant strains were inoculated on PDA and tryptone-glucose (TG) media at 28°C for 5 days, and the growth rates of the Δ*Aoste12* mutants were remarkably lower than WT strains on the two media (Fig. 1A and B). Furthermore, when cultured on a medium containing osmotic and oxidant agents, the sensitivity of the Δ*Aoste12* mutants was impaired. Notably, under the oxidative stress reagent containing menadione, the relative growth inhibition (RGI) values of the Δ*Aoste12* mutants were decreased by 27.05 to 36.81% compared to the WT strain, while under the condition of osmotic agents containing sorbitol, the RGI values were increased by 3.96 to 19.48%. At the same time, it was found that, under the conditions of 5 mM H_2_O_2_ and 0.1 M NaCl, the mycelial growth rate of the Δ*Aoste12* mutant was promoted (Fig. 1C and D).

### AoSte12 regulates the morphology of conidiophores and conidia, the conidial yield, and the transcription of sporulation-related genes.

**FIG 2:**
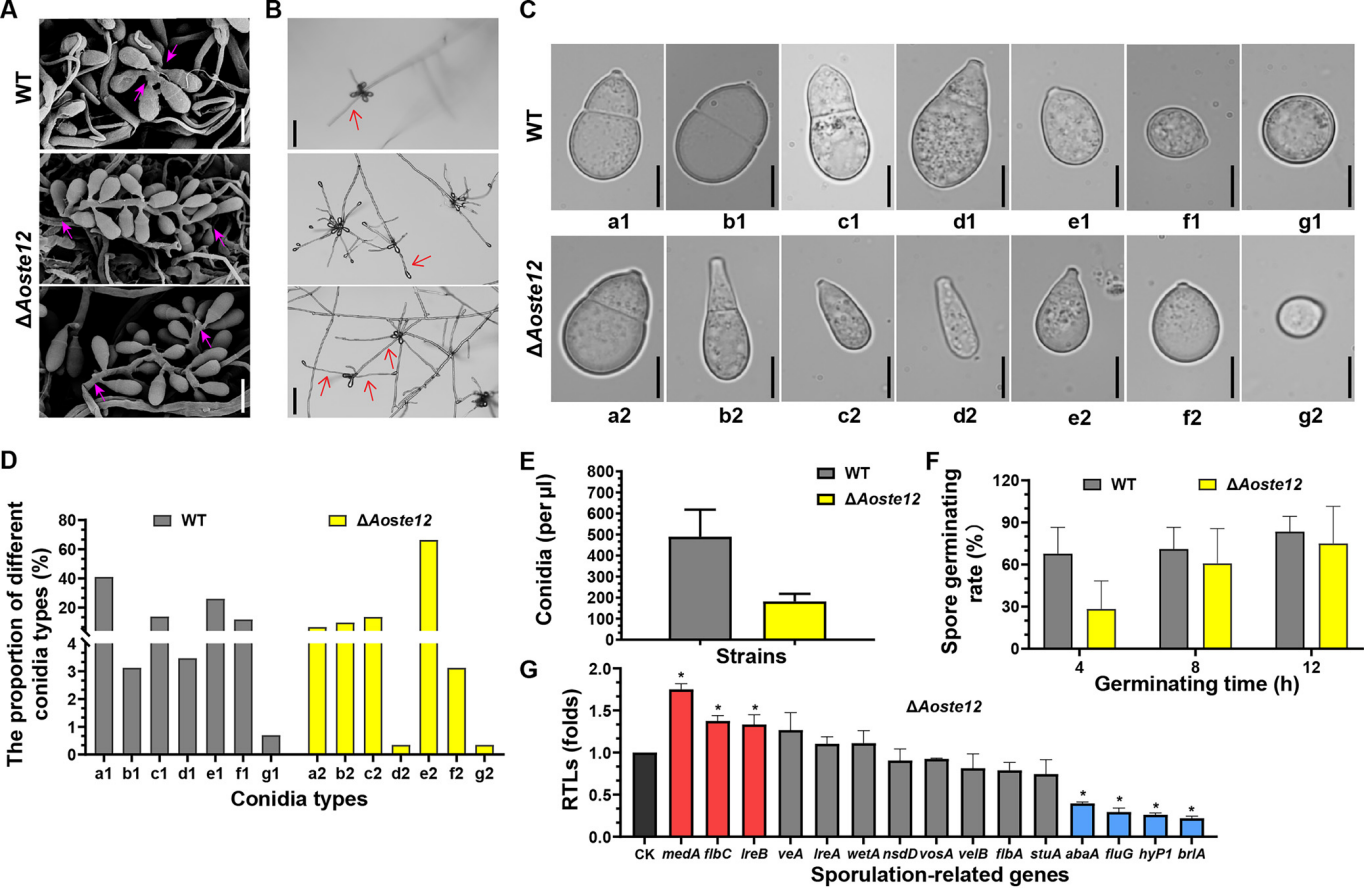
Comparison of conidiophore and conidiation in WT and Δ*Aoste12* mutant. (A) Conidiophore morphologies observed via SEM. Scale bar, 20 μm. The pink arrows indicate conidia attached on conidiophore. (B) Morphologies of spore germination on the conidiophores of WT and Δ*Aoste12* mutant. Scale bar, 100 μm. The red arrow indicates the germinating hyphae on the conidiophore. (C) Different spore morphologies of WT (a1 to g1) and Δ*Aoste12* mutant (a2 to g2). Scale bar, 10 μm. (D) Statistical analysis of the proportion of different conidia types of WT and Δ*Aoste12* mutant. (E) The conidial yields of WT and Δ*Aoste12* mutant strains. (F) Comparison of the spore germination of WT and Δ*Aoste12* mutant at 4, 8, and 12 h. (G) The relative transcription levels (RTLs) of sporulation-related genes between WT and Δ*Aoste12* mutant at 5 days. CK is the standard (which has an RTL of 1) for statistical analysis of the RTL of each gene in the deletion mutant compared to that in the WT strain under a given condition. Error bars represent the standard deviations. An asterisk indicates a significant difference between the mutant and the WT strain (Tukey’s HSD, *P < *0.05).

The deletion of *Aoste12* caused remarkable defects in the morphologies of conidiophores and conidia. When the WT and Δ*Aoste12* mutant strains were cultured on cornmeal-molasses-yeast (CMY) medium for 7 days, the conidia of the Δ*Aoste12* mutant were not attached to the top of the conidiophores as in the WT strains, but conidia were attached to the conidiophores in a string shape (Fig. 2A). In addition, after 14 days of incubation, the WT conidia had germinated on the conidiophore, whereas the conidia of the Δ*Aoste12* mutant germinated and then formed conidiophores and conidia again (Fig. 2B). Furthermore, multiple morphologies (a to g) of conidia were observed in the WT and mutant strains; 66.3% of the conidia of the Δ*Aoste12* mutants were of type e2, which denotes the immature spore without a septum, whereas most of the WT conidia were of type a1 (40%), and only 6.6% of conidia were observed in the Δ*Aoste12* mutants to be in the form of type a1 (Fig. 2C and D). At the same time, the number of conidia of the Δ*Aoste12* mutant was significantly reduced (*P < *0.05) by 62.8% compared to the WT strains, and the spore germination rate was decreased by 58.2% at 4 h, 14.3% at 8 h, and 10% at 12 h (Fig. 2E and F). In addition, the transcription of 15 genes associated with sporulation was detected via real-time quantitative PCR (RT-qPCR) analysis. The disruption of the *Aoste12* gene led to upregulated expression of *medA*, *flbC*, and *lreB*, whereas the expression levels of *abaA*, *fluG*, *hyp1*, and *brlA* were significantly downregulated (*P < *0.05) (Fig. 2G).

### AoSte12 regulates hyphal fusion, mycelial networks, and trap morphogenesis.

**FIG 3:**
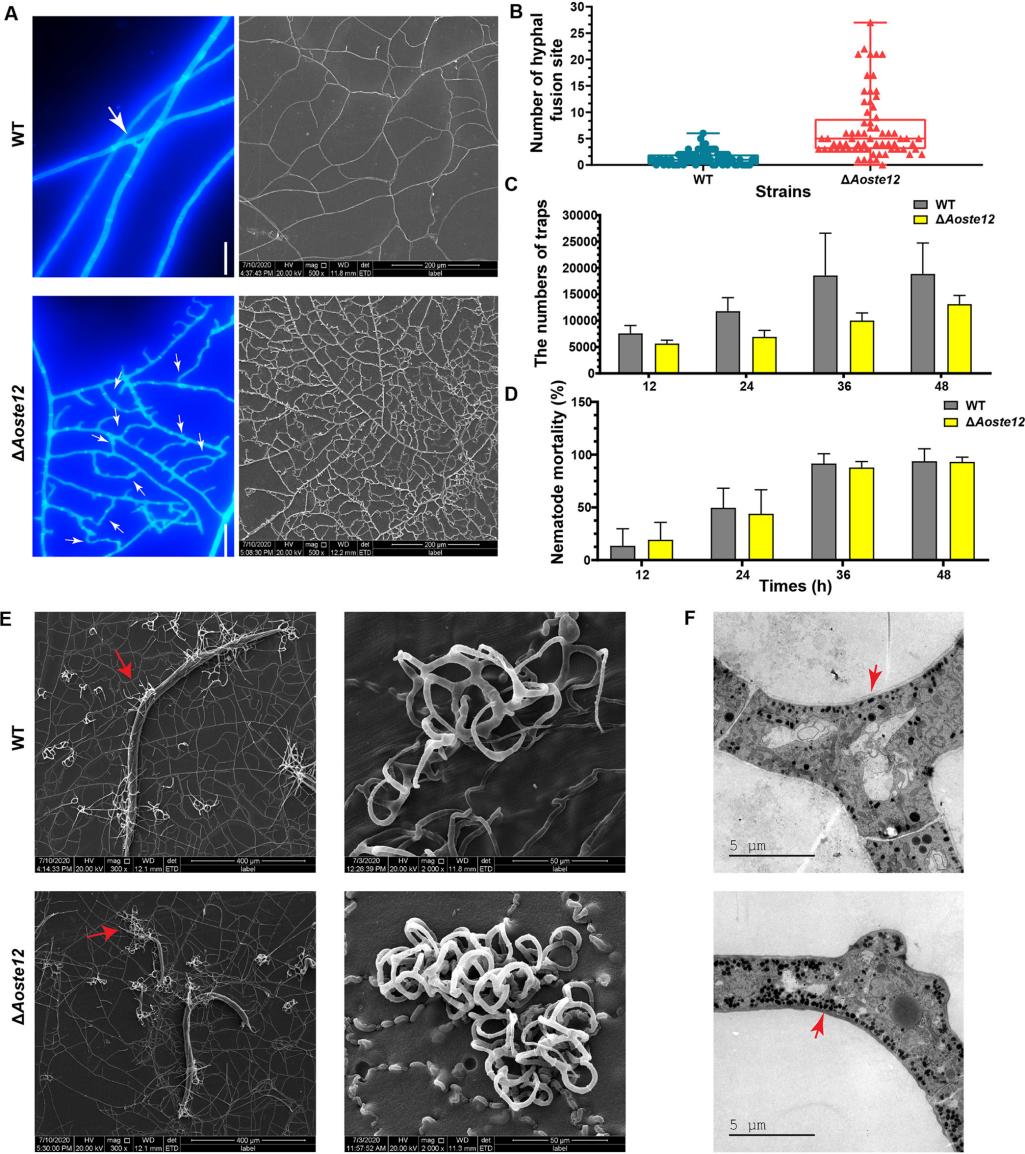
Comparison of hyphal fusion, mycelial networks, trap formation and morphologies, electron-dense bodies, and nematicidal activity of wild-type (WT) and Δ*Aoste12* mutant strains. (A) Comparison of hyphal fusion and mycelial networks of WT and Δ*Aoste12* mutant. Hyphae were stained with calcofluor white and observed via fluorescent electron microscopy; mycelial networks were observed using SEM. White arrows: hyphal fusion sites. Scale bar, 10 μm. (B) Comparison of the number of hyphal fusion sites in WT and Δ*Aoste12* mutant. Hyphal fusion sites were counted under 68 fields viewed under a microscope. (C) Comparison of traps produced by WT and Δ*Aoste12* mutant. (D) Comparison of nematode mortality caused by WT and Δ*Aoste12* mutant. Error bars in panels B to D represent the standard deviations. (E) The traps and captured nematodes of the WT and Δ*Aoste12* mutant were observed via SEM. Red arrow, the captured nematodes. (F) Comparison of electron-dense bodies in traps of WT and Δ*Aoste12* mutant through TEM. Red arrow, electron-dense bodies.

The WT and Δ*Aoste12* mutant strains were cultured on CMY medium at 28°C for 5 days, and the mycelia were stained using calcofluor white. The mycelial networks of the Δ*Aoste12* mutant became denser due to a remarkable increase in hyphal fusion (Fig. 3A). The average number of sites where hyphal fusion occurred was 7 in the Δ*Aoste12* mutants, whereas only 1.4 was observed in the WT strain (Fig. 3B). Similarly, the mycelial branching of the Δ*Aoste12* mutant was remarkably increased compared to the WT strain (Fig. 3A). To observe trap formation, the spores of the WT and mutant strains were incubated on water agar (WA) medium and cultured for 4 days; nematodes were then added for trap induction. The number of traps produced by the Δ*Aoste12* mutant were decreased compared to the WT strains, but the nematode predation efficiency was not correspondingly reduced (Fig. 3C and D). Moreover, the trap morphology was observed using scanning electron microscopy (SEM); the traps of the Δ*Aoste12* mutant contained more hyphal rings compared to the WT strain, which indicated that hyphal fusion was also increased in the traps of the Δ*Aoste12* mutant (Fig. 3E). Transmission electron microscope (TEM) observation of the traps showed that electron-dense bodies were increased in the Δ*Aoste12* mutant (Fig. 3F).

### AoSte12 regulates hyphal polar growth, the number of nuclei, and LD accumulation.

**FIG 4:**
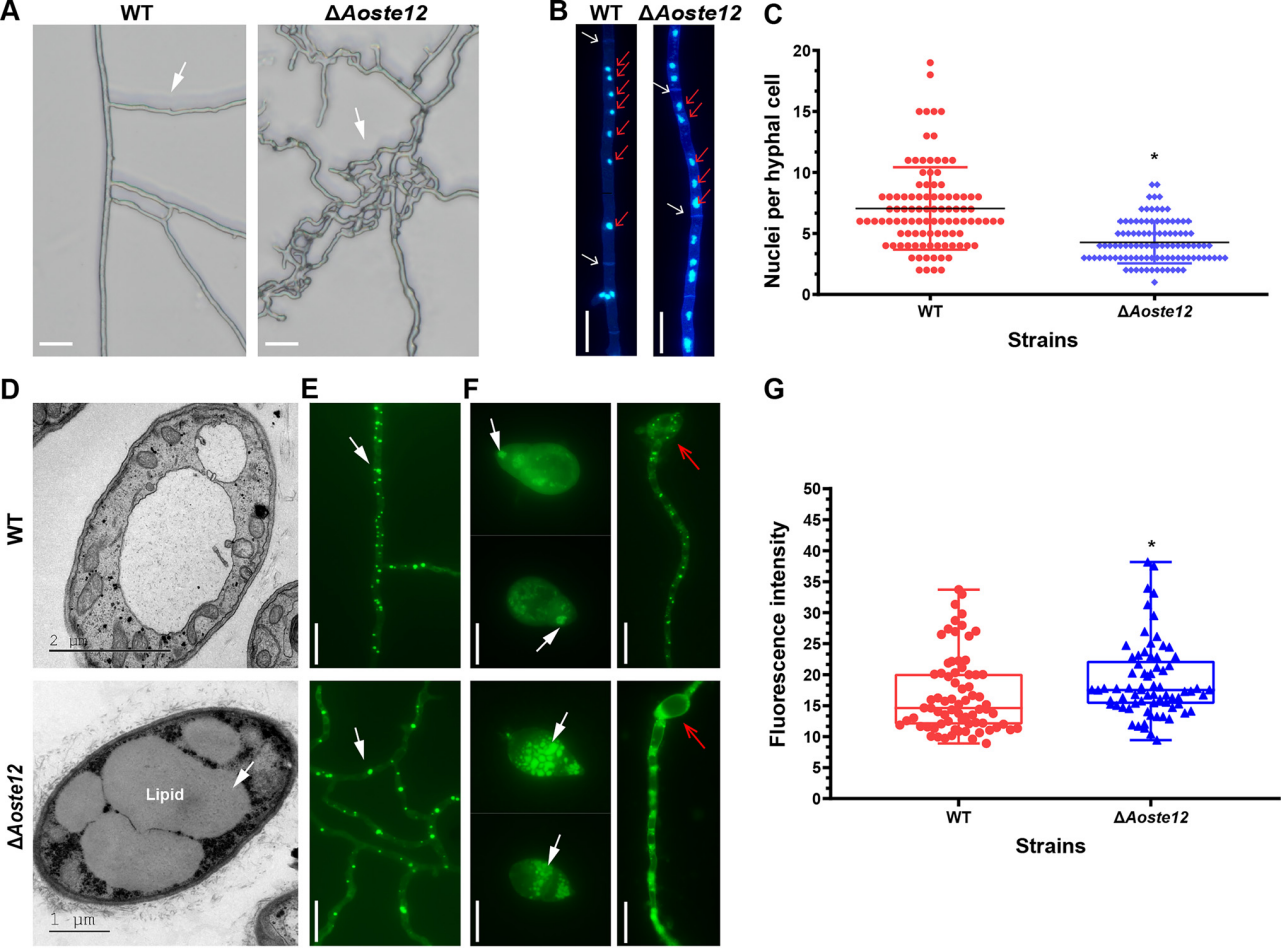
Comparison of hyphal polar growth, and nucleus and lipid droplet accumulation of wild-type (WT) and Δ*Aoste12* mutant strains. (A) Comparison of hyphal polar growth of WT and Δ*Aoste12* mutant. (B) Hyphae were stained with DAPI, and the nuclei of the WT and the Δ*Aoste12* mutant were observed. White arrow, hyphal septum; red arrow, nucleus. (C) Comparison of cell nuclei in hyphae of the WT and Δ*Aoste12* mutant. (D) Observation of cellular ultrastructure in hyphae of WT and Δ*Aoste12* mutant via TEM. (E) Observation of lipid droplets in hyphae of WT and Δ*Aoste12* mutant by staining with boron dipyrromethene dye. (F) Observation of lipid droplets in conidia and germinated hyphae of spores in WT and Δ*Aoste12* mutant. White arrow (D to F), lipid droplets. Red arrow, germinated conidium. (G) Comparison of lipid droplet content of WT and Δ*Aoste12* mutant, which were detected as fluorescence intensity, and determined for at least 70 fields observed under fluorescence electron microscopy. An asterisk indicates a significant difference between the mutant and the WT strain (Tukey’s HSD, *P < *0.05). White arrow (D, E, and F), lipid droplet. Scale bar (A, B, E, and F), 10 μm.

As mentioned above, the mycelial branch of the Δ*Aoste12* mutant was increased compared to the WT strain. When the WT and Δ*Aoste12* mutant strains were inoculated on WA at 28°C for 3 days, the polar growth of the Δ*Aoste12* mutant was altered, and the growth direction of its mycelium became confused (Fig. 4A). A further comparison of the number of nuclei in the mycelia showed that the average number of nuclei of the WT strains was seven, and that of the Δ*Aoste12* mutant was four (Fig. 4B and C). In addition, the lipid droplets (LDs) in the mycelia of the WT and mutants were observed using a BODIPY staining assay. More LDs were observed in the mycelia of the Δ*Aoste12* mutant, and the volume of the LDs became larger than that of the WT strain (Fig. 4D, E, and G). Similarly, LD accumulation in the conidia of the Δ*Aoste12* mutant was also increased, the vacuoles in the mycelia that germinated from conidia were larger in the Δ*Aoste12* mutant, and there was no accumulation of LDs when the conidia germinated for 12 h (Fig. 4F).

### Transcriptomic insight into the regulatory role of AoSte12.

**FIG 5:**
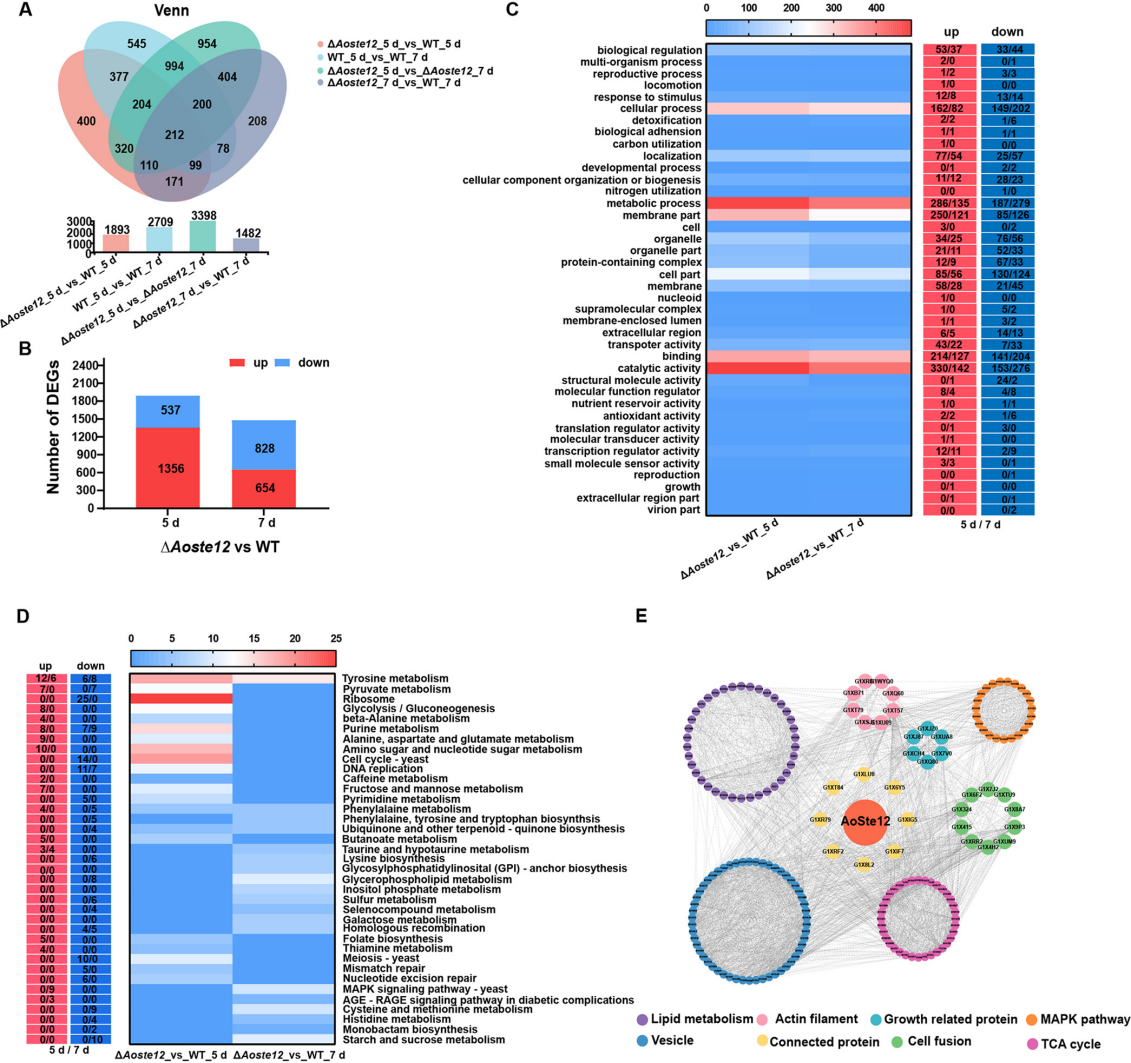
Comparison of DEGs between wild-type (WT) and Δ*Aoste12* mutant strains. (A) Venn diagram analysis of DEGs at two time points of Δ*Aoste12* mutant versus WT. (B) Numbers of upregulated and downregulated DEGs in the Δ*Aoste12* mutant versus the WT strain at different time points. (C) Gene ontology (GO) enrichment analysis of the Δ*Aoste12* mutant versus the WT strain at different time points. The red column indicates upregulated DEGs, the blue column indicates downregulated DEGs, and number/number indicates the DEGs of the Δ*Aoste12* mutant versus the WT strain at 5 and 7 days. (D) KEGG enrichment analysis of DEGs in the Δ*Aoste12* mutant versus the WT strain at different time points. (E) Proteins interact with enriched AoSte12 to correlate with the phenotypes and were analyzed through protein-protein interaction networks (STRING) and further visualized and analyzed with Cytoscape.

For further exploration into the regulatory role of AoSte12, the mycelial samples of WT and Δ*Aoste12* mutant strains were collected at 5 and 7 days postincubation (dpi) for transcriptome sequencing (RNA-Seq) analysis (see Table S2). Compared to the WT strain, the Δ*Aoste12* mutant shared 1,893 and 1,482 differentially expressed genes (DEGs) at 5 and 7 dpi, respectively (Fig. 5A). At 5 dpi, the Δ*Aoste12* mutant had 537 downregulated DEGs and 1,356 upregulated DEGs. At 7 dpi, there were 828 downregulated DEGs and 654 upregulated DEGs. Gene ontology (GO) enrichment analysis was carried out for these DEGs at 5 and 7 dpi (Fig. 5B). The upregulated and downregulated DEGs at 5 and 7 dpi displayed strong consistency, and they were mainly involved in biological regulation, cellular processes, localization, metabolic processes, membranes, membrane parts, cell parts, transport activity, binding, catalytic activity, organelles, and organelle parts (Fig. 5C).

Similarly, a *Kyoto Encyclopedia of Genes and Genomes* (KEGG) enrichment analysis was carried out; the upregulated and downregulated DEGs at 5 and 7 dpi were enriched in different pathways. At 5 dpi, the upregulated DEGs were enriched in glycolysis/gluconeogenesis and multiple metabolic pathways, such as tyrosine metabolism; taurine and hypotaurine metabolism, pyruvate metabolism; purine metabolism; alanine, aspartate, and glutamate metabolism; amino sugar and nucleotide sugar metabolism; and fructose and mannose metabolism. The downregulated DEGs were remarkably enriched in ribosomes, the cell cycle, DNA replication, meiosis, mismatch repair, and nucleotide excision repair (Fig. 5D). At 7 dpi, the upregulated DEGs were enriched in tyrosine metabolism, taurine and hypotaurine metabolism, and the MAPK signaling pathway, while the downregulated DEGs were enriched in multiple metabolism and biosynthesis, such as pyruvate metabolism, phenylalanine metabolism, cysteine and methionine metabolism, glycerophospholipid metabolism, lysine biosynthesis, and ubiquinone and other terpenoid-quinone biosynthesis (Fig. 5D).

Combining the phenotypic and transcriptome analyses of the WT and Δ*Aoste12* mutant strains, proteins interact with AoSte12 were analyzed through protein-protein interaction networks (STRING) (see Table S3), we found that AoSte12 regulates multiple cellular processes, including lipid metabolism, the tricarboxylic acid (TCA) cycle, growth, vesicle transport, actin filament, the MAPK pathway, and cell fusion (Fig. 5E). Remarkably, phospholipases PldA (AOL_s00215g30) and PlaA (AOL_s00210g100), and UBX domain protein Ubx5 (AOL_s00215g489) related to lipid metabolism and fusion were enriched. SNARE domain protein (AOL_s00188g92) and Sec22 (AOL_s00076g350) associated with vesicle transport and membrane fusion were also enriched. In addition, several signaling proteins involved in mycelia growth, conidiation, polarized growth, and trap formation were enriched, including G protein complex alpha subunit GpaB (AOL_s00109g19), MAPK kinase kinase Ssk2 (AOL_s00006g209), cell division control protein Cdc42 (AOL_s00043g439), and protein kinase regulator Ste50 (AOL_s00004g530).

### DAP-Seq analysis of AoSte12.

**FIG 6:**
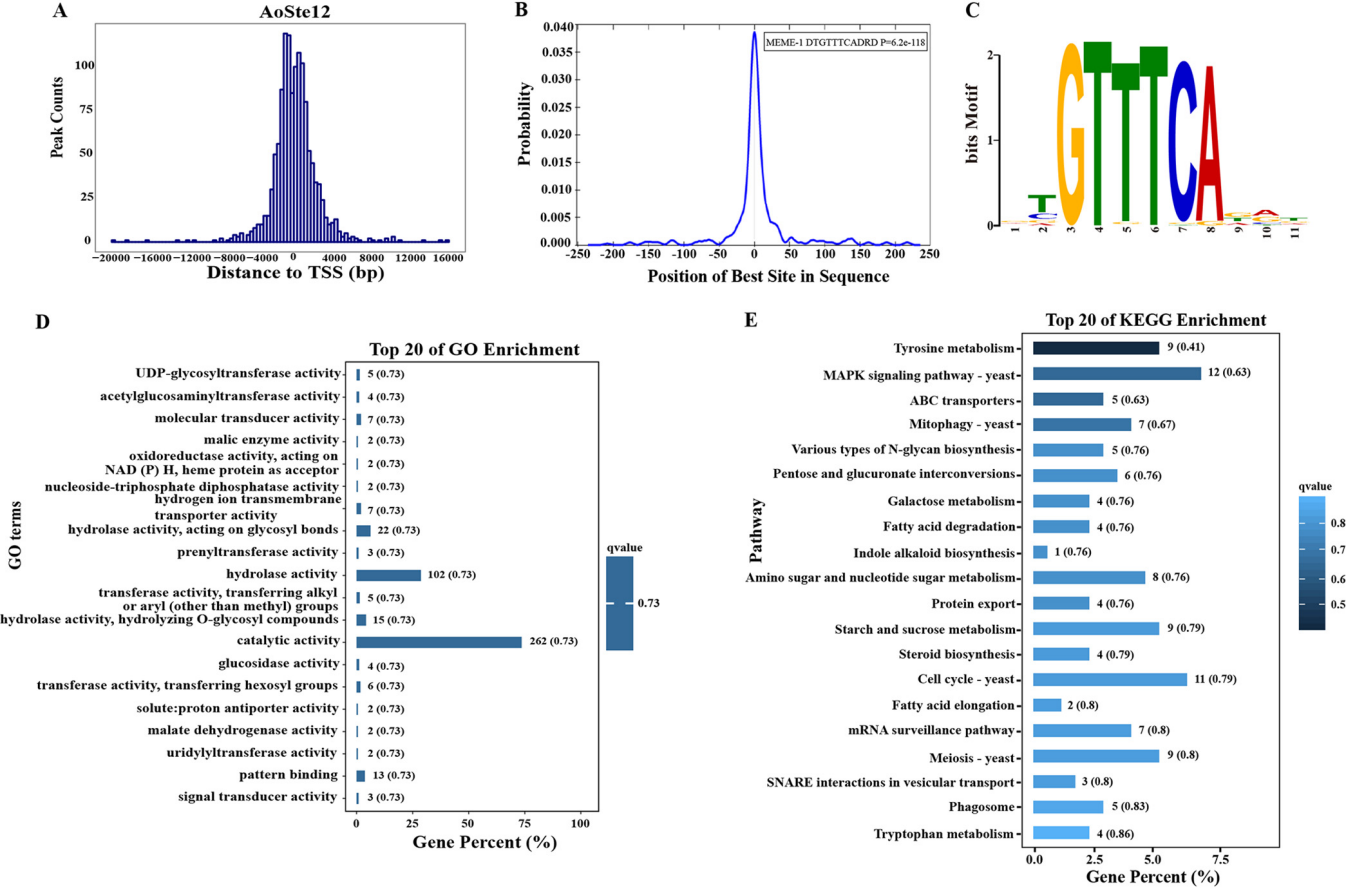
DAP-Seq analysis of AoSte12 in *A. oligospora*. (A) Genomic regional distribution of AoSte12 binding sites. TSS, transcriptional start site. (B) Positional distribution of motifs enriched significantly in the AoSte12 target peaks. (C) Motif base sequence specifically bound by AoSte12. (D) Top 20 GO enrichment terms of AoSte12 target genes. (E) Top 20 KEGG enrichment pathways of AoSte12 target genes.

The downstream target genes of *Aoste12* were analyzed using DAP-Seq; this method can be used as a partial substitute for the ChIP (chromatin immunoprecipitation) method to study transcription factor binding sites for nonmodel species (20). The distance between the peak summit and the transcriptional start site (TSS) was analyzed (Fig. 6A; see also Table S4); the distance from peak summit to the TSS was within 3 kb, accounting for about 85% of the total number of peaks (see Fig. S3). The consensus DNA binding motif of AoSte12 is “DTGTTTCADRD” (E value = 3.2E–154), and this was significantly enriched in the central region of the analyzed peak (*P* = 6.2E–118) (Fig. 6B and C). GO and KEGG enrichment analyses were further carried out on the target genes predicted by DAP-Seq. In the GO enrichment, the top 20 terms were significantly enriched in catalytic activity, hydrolase activity, and pattern binding (Fig. 6D). The top 20 KEGG pathways were enriched for the MAPK signaling pathway and autophagy processes, including mitophagy, phagosome, and SNARE interactions in vesicular transport, the cell cycle, meiosis, and fatty acid degradation and elongation (Fig. 6E).

### Comprehensive transcriptomic and DAP-Seq analysis.

**FIG 7:**
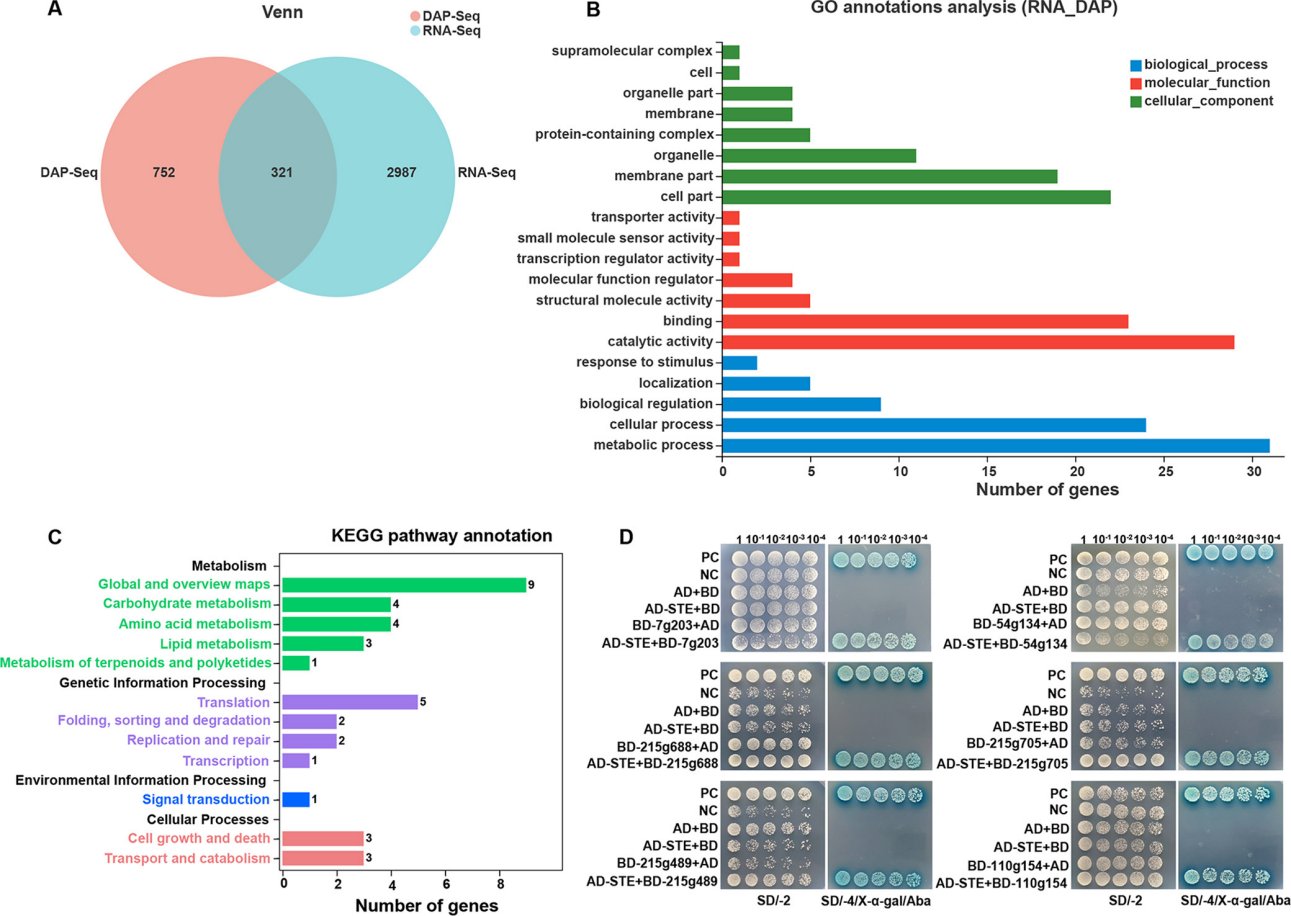
Function analysis of AoSte12 by combining transcriptome and DAP-Seq. (A) Venn diagram analysis of RNA-Seq and DAP-Seq data. RNA-Seq indicates transcriptomic data. (B) GO annotation of RNA-Seq and DAP-Seq data. (C) KEGG pathway annotation of RNA-Seq and DAP-Seq data. (D) Yeast two-hybrid (Y2H) assay of AoSte12 and six putative target proteins (AOL_s00007g203, AOL_s00054g134, AOL_s00215g688, AOL_s00215g705, AOL_s00215g489, and AOL_s00110g154) in *A. oligospora*. Plasmids pGBKT7-53 and pGADT7-T served as positive controls (PCs), whereas pGBKT7-Lam, pGADT7-T, pGBKT7, and pGADT7 served as negative controls (NCs). Yeast transformants were diluted in 0.9% NaCl, and on this basis, diluted four times with equal volume for 10^0^, 10^−1^, 10^−2^, 10^−3^, 10^−4^. Growth was determined on SD/–Trp/–Leu (SD/-2), SD/–Trp/–Leu/–His/–Ade (SD/-4), and SD/-4/X-α-Gal/Aba media with serially diluted yeast cells.

In order to further explore the function of AoSte12, transcriptomic and DAP-Seq data were comprehensively analyzed. First, the transcriptomic and DAP-Seq data were screened, 3,308 DEGs of the transcriptome were obtained, and 1,073 genes were selected due to the promoter of the target gene being located in the 2-kb sequence range upstream of the transcription start site. Venn analysis showed that there were 321 genes in both data sets (Fig. 7A). Then, GO and KEGG enrichment analyses showed that these genes were involved in multiple biological processes and metabolic pathways. In the GO analysis, cell parts, membrane parts, and organelles were enriched in the cellular component; catalytic activity and binding were enriched in molecular function; and metabolism and cellular processes were enriched in biological processes. In the KEGG pathway annotations, global and overview maps, carbohydrate metabolism, amino acid metabolism, lipid metabolism, translation, cell growth and death, and transport and catabolism were enriched (Fig. 7B and C).

### Y2H analysis.

According to the phenotypic differences between the WT and the Δ*Aoste12* mutant strains, several genes were screened and verified using Y2H, and a total of six proteins interacting with AoSte12 were verified, including UDP-glycosyltransferase (AOL_s00007g203), malate dehydrogenase (AOL_s00054g134), vacuolar sorting protein Pep3 (AOL_s00215g688), ABC-type transporter (AOL_s00215g705), UBX domain-containing protein Ubx5 (AOL_s00215g489), and MAK-2 orthologue protein Fus3 (AOL_s00110g154) (Fig. 7D).

### AoSte12 regulates secondary metabolites.

**FIG 8:**
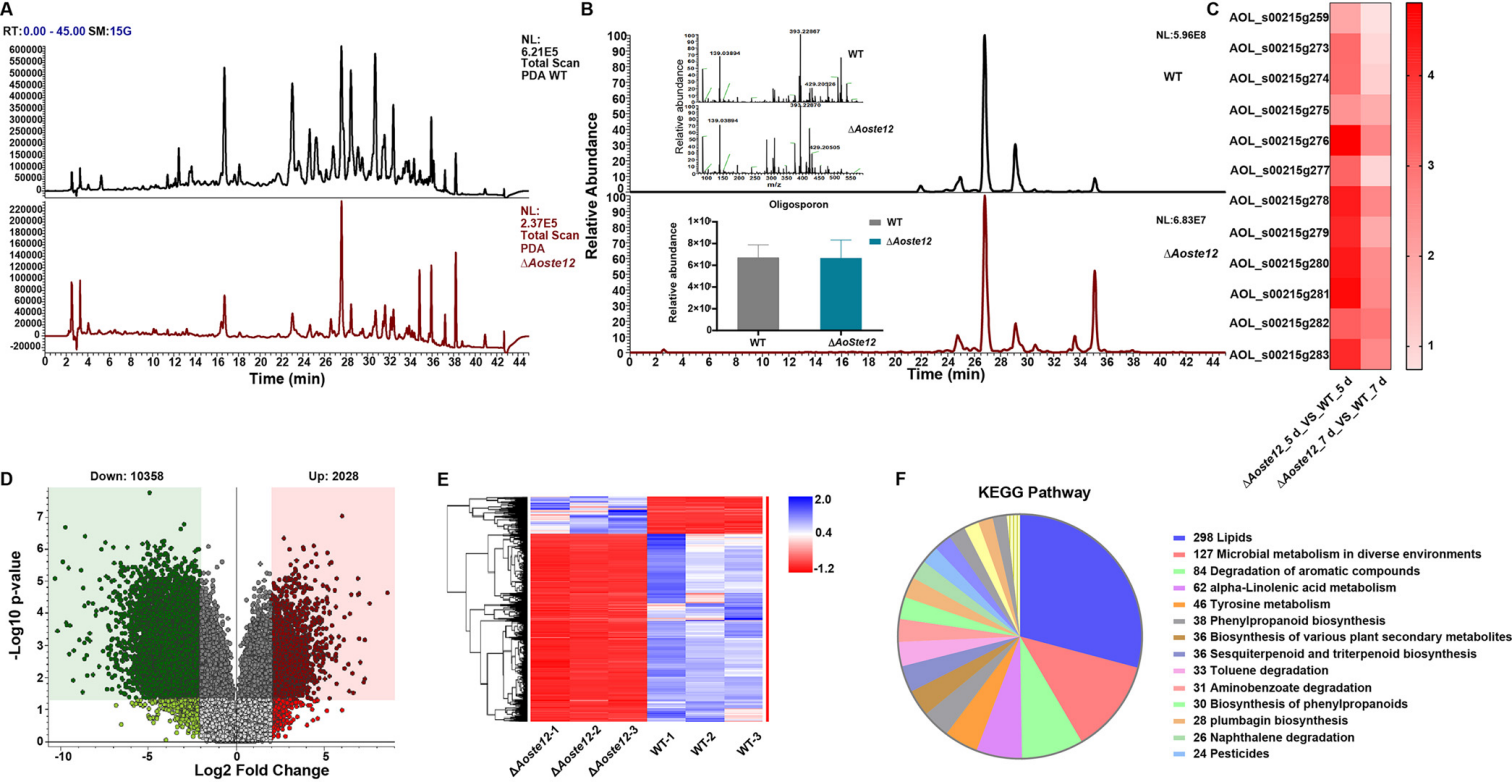
Comparison of metabolic profiling between wild-type (WT) and Δ*Aoste12* mutant strains. (A) Comparison of high-performance liquid chromatography (HPLC) profiles of the WT and Δ*Aoste12* mutant strains. PDA, photo-diode array. (B) Comparison of chromatogram of arthrobotrisin anion peaks of WT and Δ*Aoste12* mutant strains. Arthrobotrisins were verified via diagnostic ion fragments at *m/z* 139, 393, and 429 through a mass spectrogram. The histogram showed that the arthrobotrisin content was comparable to the peak area of the WT and Δ*Aoste12* mutant strains. (C) A heatmap shows the relative transcription levels of genes associated with the biosynthesis of arthrobotrisins in the Δ*Aoste12* mutant versus the WT strain at different time points. (D) Volcano plot of differential metabolites between WT and Δ*Aoste12* mutants. (E) Heatmap of upregulated and downregulated metabolic pathways between Δ*Aoste12* mutant and WT strain determined via KEGG enrichment. (F) Numbers of KEGG pathways in the Δ*Aoste12* mutant versus the WT strain.

The extracts of the WT and Δ*Aoste12* mutant strains were determined via liquid chromatography-mass spectrometry (LC-MS) (see Table S5). The kurtosis values of the WT and Δ*Aoste12* mutant strains were compared through the chromatogram. Compared to the WT strain, the kurtosis of the Δ*Aoste12* mutant showed a downward trend over a 10- to 38-min retention period (Fig. 8A). Furthermore, the specific compound arthrobotrisins identified from *A. oligospora* and other NT fungi were analyzed, and their contents showed no differences between the WT and Δ*Aoste12* mutant strains (Fig. 8B), whereas the transcriptional levels of genes associated with the biosynthesis of arthrobotrisins were remarkably upregulated in the Δ*Aoste12* mutant compared to the WT strain (Fig. 8C).

An analysis of the metabolic data revealed that 10,358 compounds were downregulated and 2,028 compounds were upregulated in the Δ*Aoste12* mutant compared to the WT strain (Fig. 8D). Similarly, most of the enriched metabolic pathways were downregulated in the Δ*Aoste12* mutant (Fig. 8E). In addition, KEGG enrichment analysis of these metabolic pathways revealed that several pathways were highly enriched, such as the LD accumulation-related metabolic pathway, microbial metabolism in diverse environments, the degradation of aromatic compounds, alpha-linolenic acid metabolism, the biosynthesis of various plant secondary metabolites, sesquiterpenoid and triterpenoid biosynthesis, and toluene degradation (Fig. 8F).

## DISCUSSION

Ste12 is a conserved transcription factor in fungi and plays an important regulatory role in mating, hyphal growth, conidiation, sexual development, hyphal fusion, and pathogenicity (26–31). In this study, we characterized an orthologue, Ste12, in the NT fungus *A. oligospora* using a combined analysis of multiple phenotypes and multi-omics. Our results show that AoSte12 plays an important role in mycelial growth, stress resistance, conidiation, hyphal fusion, trap morphogenesis, nucleus, LD accumulation, and secondary metabolism. Meanwhile, transcriptomic and DAP-Seq analyses also confirmed that AoSte12 is involved in the regulation of diverse biological processes.

The disruption of *Aoste12* affects mycelial growth and stress resistance in *A. oligospora*. Similarly, the deletion of the *ste12* gene results in the slow growth rate of mycelia in N. crassa (33), the NT fungus *Drechslerella dactyloides* (34), and the plant-pathogenic fungus *Setosphaeria turcica* (35). However, the absence of orthologs of *ste12* has no influence on mycelial growth in several filamentous fungi, including Fusarium graminearum (36), Aspergillus nidulans (37), and *M. grisea* (30). Moreover, Δ*ste12* mutant had the same sensitivity to H_2_O_2_ with WT strain in Alternaria alternata (38), while the Δ*Aoste12* mutant had lower sensitivity to oxidation reagents (H_2_O_2_ and menadione) than WT strain in *A. oligospora*. Accordingly, the responses to stimuli and antioxidant activity were enriched in GO terms. Therefore, Ste12 plays a varied role in the mycelial growth of different fungal species, and AoSte12 also plays a minor role in stress resistance for *A. oligospora*.

Conidia are special reproductive propagules with which filamentous fungi can expand their reproduction when their living conditions are no longer fit for apical extension growth (39). The deletion of *ste12* in F. graminearum and *M. grisea* had no effect on the formation and germination of conidia (30, 36). In *D. dactyloides*, the Δ*Ddste12* mutant had defects for conidiation, and the number of conidia decreased by only 19.7% compared to the WT strains (34). The absence of Ste12 caused conidia to be produced only under the condition of 0.4 M KCl in the growth medium, in Metarhizium *rileyi* (40). Similarly, conidia yield was decreased in the Δ*ste12* mutant of *S. turcica* (35), and A. oryzae (25). In this study, the conidia yield of the Δ*Aoste12* mutant was significantly reduced compared to the WT strain, and the morphology of the conidiophore of the Δ*Aoste12* mutant was also remarkably changed. Moreover, the morphology of the conidia of the Δ*Aoste12* mutant was mostly immature with no septum, and the spore germination rate was remarkably reduced. In addition, the transcription of genes related to conidiation was also altered, especially the genes encoding regulators *abaA*, *brlA*, and *fluG*, which coordinate conidiation-specific gene expression (39, 41), the expressions of which were remarkably downregulated in the Δ*Aoste12* mutant. Therefore, Ste12 plays a conserved role in sporulation in many fungi, and AoSte12 also plays an important role in regulating the development of conidiophores.

The mycelium network structure formed by hyphal fusion in filamentous fungi is crucial for the organization and function of colonies (42). N. crassa is a model fungus for studying hyphal fusion, and the *ste12* homologous protein gene *pp-1* is essential for conidial anastomosis tubes and hyphal fusion (22). The Δ*ste12* mutant in T. atroviride has the same phenotype as the Δ*pp-1* mutant in N. crassa, whereas in A. oryzae, Ste12 plays a negative regulatory role in hyphal fusion (24, 25). However, in *E. festucae* and F. oxysporum, the deletion of *ste12* has little effect on hyphal fusion (27, 43). In this study, the deletion of *Aoste12* remarkably promoted the frequency of hyphal fusion, and the Δ*Aoste12* mutant showed more dense mycelial networks. Therefore, orthologous Ste12 plays a divergent role in hyphal fusion in diverse fungi, and plays a role in negative regulation for hyphal fusion and the formation of mycelial networks in *A. oligospora*.

In addition, Ste12 is involved in virulence and is necessary for Candida glabrata to produce pseudomycelia in nitrogen-deficient cultures (44). Similarly, Ste12 is essential for pathogenicity in several pathogenic fungi, such as F. graminearum (36), F. oxysporum (26), *M. grisea* (30), *M. rileyi* (40), and *S. turcica* (35). Recently, orthologous Ste12 was identified in the two NT fungi *D. dactyloides* and *A. oligospora* (strain TWF154); disruption of the *Ddaste12* gene in *D. dactyloides* disabled the cell inflation of constricting rings and led to an inability to capture nematodes (34). In *A. oligospora* (strain TWF154), the Δ*ste12* mutant did not form traps when exposed to nematodes for 24 h; after prolonged incubation in the presence of nematodes, the Δ*ste12* mutant developed a few traps with abnormal morphology (32). In this study, the deletion of *Aoste12* resulted in a reduction in trap formation, whereas the traps of the Δ*Aoste12* mutant consisted of more hyphal loops, which may remedy the reduced trap formation. Thus, there is no obvious difference between nematode predation ability in the WT and Δ*Aoste12* mutant strains. Overall, these results show that Ste12 has a conserved role in fungal pathogenicity, and it plays a crucial role in the regulation of trap morphogenesis for NT fungi.

Filamentous fungi are a kind of fungi that show continuous growth at the tip of the growing hypha. Polar growth requires the continuous supply of proteins and LDs to the top of the hypha. This transportation is achieved by vesicles through the actin and microtubule cytoskeleton (45). In *A. oligospora*, the disruption of *Aoste12* impaired the polar growth of hypha and affected the formation of hyphal networks, which caused the Δ*Aoste12* mutant to grow in a confused direction and increased the density of mycelial networks. Furthermore, BODIPY fluorescent staining showed that there were more LDs in the Δ*Aoste12* mutant, and the number of nuclei of the Δ*Aoste12* mutant was reduced. These results show that AoSte12 has a negative effect on the polar growth of hyphae, and it also plays an important role in regulating the accumulation of LDs and the number of nuclei.

In recent years, transcriptomic analysis has been broadly applied to elucidate the mechanism of fungal growth development (46–48). In this study, the absence of *Aoste12* caused 16.5 and 12.9% of genes to be differentially expressed at 5 and 7 dpi, respectively. GO enrichment analysis showed that these DEGs were mainly enriched for cellular processes, localization, metabolic processes, membranes, cell parts, binding, catalytic activity, and organelles, indicating that AoSte12 plays a multifunctional regulatory role in the mycelial growth and development process. KEGG enrichment analysis showed that the upregulated genes were remarkably focused on glycolysis/gluconeogenesis, multiple metabolic pathways, and the MAPK signaling pathway, whereas the downregulated genes were remarkably focused on ribosome, cell cycle, DNA replication and repair, meiosis, multiple metabolism, and biosynthesis processes. These pathways are closely associated with phenotypic alteration, such as mycelial growth, conidiation, the cell nucleus, and trap formation. Moreover, several genes associated with lipids, actin, growth, the MAPK pathway, vesicles, cell fusion, and TCA cycles were significantly enriched and consistent with phenotypic traits. For example, G protein complex alpha subunit and cell division control protein Cdc42 have been proven to play a crucial role in mycelial growth, conidiation, trap formation, and stress response in *A. oligospora* (11, 49). Meanwhile, Ssk2 and Ste50 are indispensable components of the MAPK signaling cascade and have been reported to be involved in growth, conidiation, stress response, and virulence in *A. oligospora* and other pathogenic fungi (32, 50). In addition, phospholipase PldA is required for polarized growth and cell fusion in *E. festucae* (51), and PlaA regulates conidiation, appressorial turgor pressure, and pathogenicity in M. oryzae (52). The ortholog of SNARE domain protein (AOL_s00188g92) mediates vesicle membrane fusion with target compartments (53), and vesicle transport protein Sec22 is involved in regulating cell wall integrity, growth, reproduction, pathogenicity, the regulation of ROS, and the expression of extracellular enzymes in filamentous fungi (54).

DAP-Seq data showed that the consensus DNA binding motif of AoSte12 was consistent with PP-1 in N. crassa (20). In the GO enrichment, catalytic activity, hydrolase activity, and hydrolase activity acting on glycolytic bonds were significantly enriched. Furthermore, tyrosine metabolism, the MAPK signaling pathway, ABC transport, mitophagy, and fatty acid degradation were enriched in the KEGG enrichment, which was consistent with the transcriptomic analysis. Combining the transcriptome with DAP-Seq for a comprehensive analysis, the GO enrichment result is consistent with the transcriptomic analysis, and the KEGG enrichment analysis indicates that AoSte12 regulates diverse metabolic pathways such as global and overview maps, carbohydrate metabolism, amino acid metabolism, lipid metabolism, translation, cell growth and death, and transport and catabolism. Based on these results, AoSte12 is involved in cellular growth and metabolic processes. Several genes involved in these processes were further verified through Y2H. In N. crassa, Ste12 is regulated by the MAPK pathway, and its ortholog of PP-1 is regulated by MAK-2 (55). Moreover, there are no potential Adv-1 binding motifs upstream of *ste12* (*pp-1*), but there are several potential Ste12 binding motifs upstream of *adv-1*, which suggested that Ste12 regulates *adv-1* and Adv-1 directly regulates downstream gene expression in N. crassa (20). However, our DAP-Seq data failed to identify AoSte12 binding to the promoter of *Aoadv-1*, and no interaction between the AoSte12 and AoAdv-1 was verified by Y2H analysis (data not shown); therefore, the interaction between the AoSte12 and AoAdv-1 remains to be further explored. In our analysis, the MAK-2 homologous protein Fus3 can interact with AoSte12, which further proves the regulatory relationship between Fus3 and AoSte12. At the same time, AoSte12 can interact with vacuolar sorting protein Pep3, UDP-glycosyltransferase, UBX domain-containing protein Ubx5, ABC-type transporter, and malate dehydrogenase. Of these, UDP glycosyltransferases are major phase II enzymes involved in the metabolism and detoxification of exogenous organisms (56), Ubx5 is related to lipid accumulation and the stress response (57), malate dehydrogenase is involved in the regulation of the TCA cycle (58), Pep3 is a low-abundance peripheral vacuolar membrane protein (59), and ABC-type transporter is involved in membrane transporters (60); they might regulate membrane transport and hyphal fusion. These results suggest that AoSte12 plays a pleiotropic role in diverse biological processes by regulating diverse targets.

Fungi are rich sources of secondary metabolites with important medicinal value, with potential uses as antibiotics, antitumor medications, and immunosuppressants (61, 62). Secondary metabolites play an important role in the development and pathogenesis of fungi, for example, the cycloundecapeptide cyclosporine was isolated from the insect-pathogenic fungus *Tolypocladium inflatum* for its antifungal activity and later developed as an immunosuppressant drug (63). The interaction between nematodes and NT fungi requires sophisticated inter-organismic communication with low-molecular-weight signaling molecules (64). The Mak-2/PP-1 regulation pathway also regulates the synthesis of secondary metabolites in N. crassa (33). In this study, compound kurtosis was significantly downregulated at multiple retention times, indicating that AoSte12 is involved in the regulation of the biosynthesis of secondary metabolites in *A. oligospora*.

**FIG 9:**
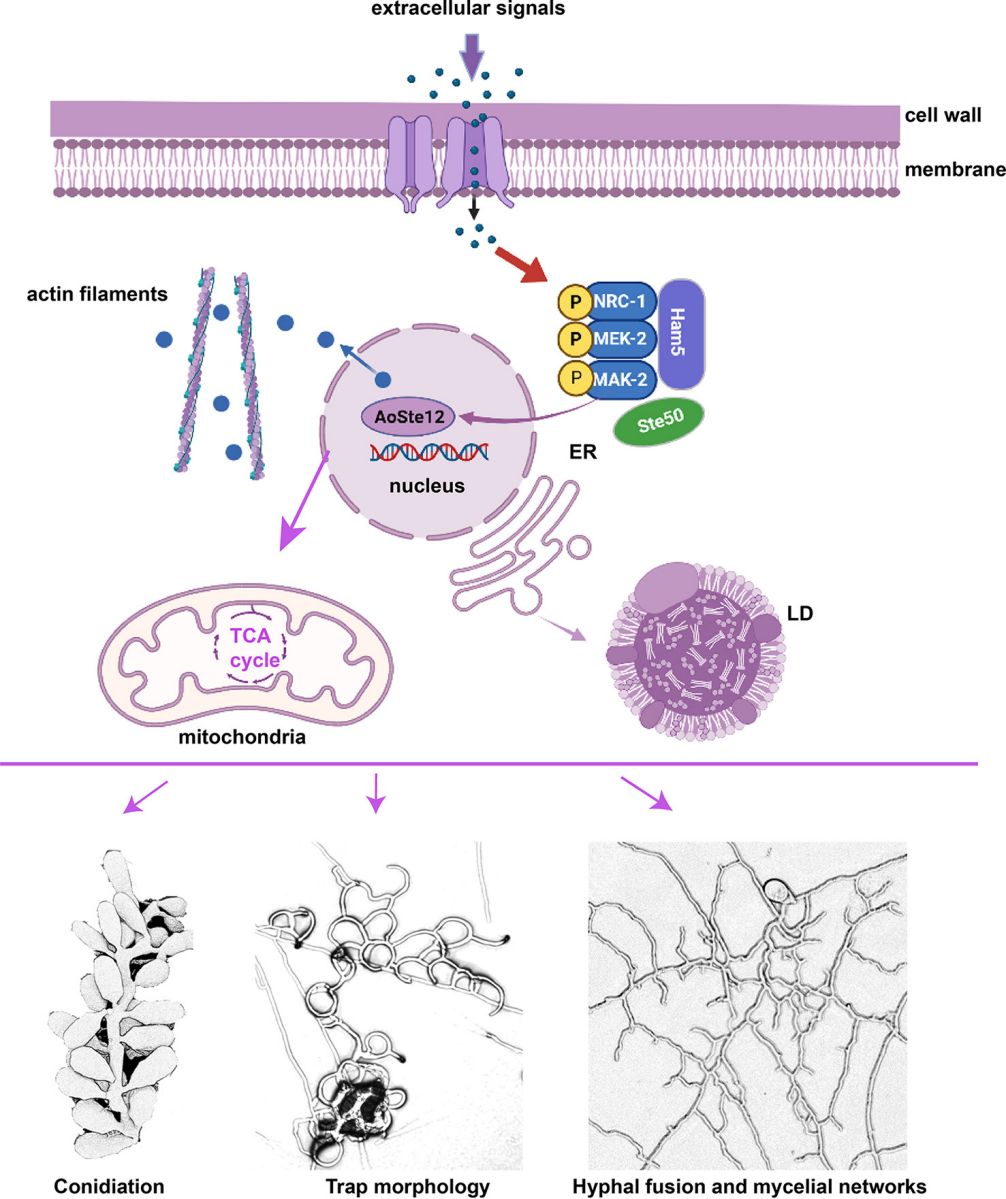
Proposed model for AoSte12 regulation in *A. oligospora*. Our findings suggest that AoSte12 is regulated by Fus3 and then regulates multiple cellular processes such as the TCA cycle, lipid metabolism, actin filament, and vesicle transport. It also plays an important regulatory role in conidiation, hyphal fusion, trap formation, and pathogenicity. ER, endoplasmic reticulum; LD, lipid droplet. This image was created with BioRender.com.

Combing the analysis of phenotypic traits, transcriptomics, DAP-Seq, and metabolic analyses between the WT and the Δ*Aoste12* mutant strains, our results show that AoSte12 plays an important regulatory role in mycelial growth, hyphal fusion, conidiation, pathogenicity, secondary metabolism, and so on. Similar to the homologous protein PP-1 in N. crassa, AoSte12 is regulated by Fus3 (ortholog of MAK-2 in N. crassa) and then regulates multiple biological processes such as the TCA cycle, lipid metabolism, actin filament, and vesicle transport (Fig. 9). However, the physiology of the Δ*Aoste12* mutant was different from that of the Δ*pp*-1 mutant in N. crassa, whose frequency of hyphal fusion was reduced. For the Δ*Aoste12* mutant, the frequency of hyphal fusion in hyphal networks and traps was significantly increased. In addition, our results show obvious differences from the phenotypic alteration (trap morphology and conidiation) of another species of *A. oligospora* (strain TWF154). These divergences may be caused by a difference between fungal species, whereas the detailed mechanism is still unknown. In summary, our results provide deep insights for revealing the function and regulatory mechanisms of AoSte12, and our results show that AoSte12 has pleiotropic roles especially in hyphal fusion, conidiation, and trap development. These results lay a foundation for further study into the function and regulatory mechanism of orthologs of Ste12 in hyphal fusion and trap formation in *A. oligospora* and other NT fungi.

## MATERIALS AND METHODS

### Strains and culture conditions.

*Arthrobotrys oligospora* (ATCC 24927) and derived mutant strains were incubated on PDA medium. PDA and TG media were used for phenotypic analyses of the WT and mutant strains, as previously described (14). S. cerevisiae (FY834) was cultured on yeast extract peptone dextrose medium for the recombinational cloning procedure, and yeast strains with the correct knockout vector were selected on SC-Ura defective medium (65). pCSN44 and pRS426 plasmids were carried by Escherichia coli strain DH5α (TaKaRa, Shiga, Japan) for hygromycin B resistance gene *hph* cloning and construction of the knockout vector, respectively. The regeneration of *A. oligospora* protoplasts was carried out on PDAS supplemented with 200 μg/mL hygromycin. Caenorhabditis elegans (strain N2) worms were cultured on an oatmeal medium at 26°C and used for bioassays.

### Sequence and conserved domain analysis of AoSte12 in *A. oligospora*.

The protein sequences of Ste12 from S. cerevisiae and N. crassa were used as a query to seek out the ortholog of Ste12 in *A. oligospora*. The homologous sequences of Ste12 from different fungi were downloaded from the NCBI database (https://www.ncbi.nlm.nih.gov/); the sequence similarity was analyzed using the DNAman software package (v5.2.2; Lynnon Biosoft, St. Louis, Canada), and the structural domains of the homologs of Ste12 were analyzed on the Pfam website (https://www.ebi.ac.uk/interpro). The amino acid sequences of Ste12 proteins were aligned with ClustalX, and the MEGA 7 software package with default parameter settings was used to construct a neighbor-joining tree (66).

### *Aoste12* deletion and verification.

The disruption of the *Aoste12* gene was performed using a modified yeast cloning procedure, as previously described (67, 68). The 5′ and 3′ flanking sequences of the *Aoste12* gene and the *hph* cassette were amplified from *A. oligospora* genome DNA and pCSN44, respectively, using the paired primers listed in Table S1. The amplified fragments and the linearized pRS426 plasmid were cotransformed into S. cerevisiae (FY834) to obtain the correct knockout vector pRS426-AoSte12-hph, and the disruption sequences were amplified from it using Ste12LF1 and Ste12RR1 and then transformed into *A. oligospora* protoplasts, as previously described (67, 68). The positive transformants were confirmed via PCR and Southern blotting, as described previously (45, 69).

### Analysis of vegetative growth and stress tolerance.

The WT and mutant strains were cultured on PDA and TG medium for observing mycelial growth. The growth rate of mycelia was recorded in 24 intervals. In order to observe the cell morphology, calcofluor white (Sigma-Aldrich, St. Louis, MO), 4′,6′-diamidino-2-phenylindole (DAPI; Sigma-Aldrich), and boron dipyrromethene (BODIPY) dye (Sigma-Aldrich) were used to stain the mycelia, as described previously (50). The mycelia were examined using transmission electron microscopy (TEM; Hitachi, Tokyo, Japan).

In addition, the TG medium was used as the base medium supplemented with different chemical reagents to test the stress tolerance; two osmotic agents (0.1, 0.2, and 0.3 M NaCl and 0.25, 0.5, and 0.75 M sorbitol) and two oxidant agents (5, 10, and 15 mM H_2_O_2_ and 0.05, 0.07, and 0.09 mM menadione) were used. The relative growth inhibition (RGI) values of the strains were calculated as previously described (10, 14).

### Observation of conidiospores, colony morphology, trap formation, and morphology.

The fungal strains were inoculated on CMY medium at 28°C for 7 days, and the conidiophores and conidia were observed using SEM (FEI Quanta-200, Hillsboro, OR) and TEM (Hitachi, Tokyo, Japan). The spores were washed from the medium, as described previously (50). A total of 2 × 10^4^ spores were spread on water agar medium. The spore germination rate was counted at 4, 8, and 12 h. Similarly, 2 × 10^4^ spores were spread on water agar medium at 28°C for 4 days. After spore germination, approximately 200 nematodes were added to each plate for the induction of trap formation. The mycelial morphology, traps, and the captured nematodes were imaged using SEM, and the intracellular structures of the traps were observed using TEM.

### Transcriptome sequencing and analysis.

The WT and mutant strains were cultured on CMY medium with cellophane at 28°C, and the mycelia were collected at 5 and 7 days postinoculation. Three independent biological replicates were used for each sample, and the samples were sent to Shanghai Meiji Biological Company (Shanghai, China) for RNA sequencing and data analysis. High-quality RNA samples were used to construct a sequencing library that was sequenced on an Illumina HiSeq 4000 platform (Illumina, San Diego, CA). An average of 43.5 million clean reads was obtained per sample. The percentage of Q30 was 93.49 to 94.21%, and the GC content was 47.82 to 48.43% (see Table S2). Principal-component analysis showed that the three repeats of the WT and mutant strains at each time stage had high similarity and correlation (see Fig. S4). Furthermore, the transcriptional levels of 12 genes were determined using RT-qPCR analysis, and the results show that all the selected genes had the same expression pattern, thus verifying the accuracy of the transcriptome data (see Fig. S5). DEGs were identified based on the thresholds of | log_2_ ratio | ≥ 1 and adjusted *P* < 0.05. The RNA-Seq data were analyzed through the OmicShare online platform.

In addition, proteins interact with AoSte12 were analyzed through protein-protein interaction networks STRING (https://cn.string-db.org/) and further visualized and analyzed with Cytoscape.

### DAP-Seq and data analysis.

The cDNA sequence encoding protein AoSte12 was constructed in the Halo Tag plasmid. The open reading frame of *Aoste12* was amplified from the cDNA of the WT strain. This was inserted into the DB3 vector with a Halo-tag, the construct vector pAoSte12-halo was identified via sequencing, and then the vector and the genome DNA of *A. oligospora* were sent to Genedenovo Biotechnology (Guangzhou, China) for DAP-Seq analysis. The AoSte12 protein binds to the target genome fragment, then the DNA fragment of the genome bound by the target protein can be enriched and the input group used as the control. In combination with high-throughput sequencing technology, DNA products after DAP were sequenced and analyzed, DNA binding sites of target proteins were searched from the whole genome, and high-throughput data results were obtained using efficient sequencing methods. The DNA libraries were sequenced on the Illumina sequencing platform by Genedenovo Biotechnology Co. The average total number of effective reads after IP group (experimental group) and input filtering was about 80 million (see Table S4). The number of bases whose quality value was above Q30 and the percentage of them in the total number of filtered effective bases was 94.12%, on average (see Table S4). The distribution of peaks on gene functional elements is provided in Table S4 in the supplemental material. MEME-ChIP (https://meme-suite.org/meme/doc/meme-chip-output-format.html) was used to detect significant motif sequences in the peak sequence.

### RT-qPCR analysis.

WT and mutant strains were cultured on CMY at 28°C for 5 day, and mycelial samples were collected for total RNA extraction according to the Axygen kit procedure (Axygen Biotech Company, Hangzhou, China) and then reverse transcribed into cDNA using a PrimeScript RT reagent kit (TaKaRa, Shiga, Japan). They were then used as the template for qPCR. Specific primers (see Table S1) to detect the transcript levels of sporulation-related genes, and the *A. oligospora* β-tubulin gene (AOL_s00076g640) was used as an internal standard using a Roche LightCycler 480 relative quantitative method (Roche Center, China). The relative transcription level (RTL) of each gene was calculated as the ratio of the transcription level between the mutant and the WT strain according to the 2^–ΔΔ^*^CT^* method (70).

### Y2H analysis.

The cDNA of *A. oligospora* was obtained as mentioned, and then the cDNA sequences of *Aoste12*, AOL_s00215g688, AOL_s00007g203, AOL_s00215g489, AOL_s00215g705, AOL_s00054g134, and Fus3 were amplified using the primers listed in Table S1. The cDNA sequence of *Aoste12* was cloned into the prey plasmid pGADT-7, and other cDNA sequences were cloned into the bait plasmid pGBKT-7. The correctly constructed vectors were cotransformed in S. cerevisiae strain Y2H Gold according to the LiAc/SS-DNA/PEG transformation protocol (TaKaRa, Dalian, China). The transformants were spread on SD/–Trp/–Leu and SD/–Trp/–Leu/–His/–Ade medium and verified using SD/–Trp/–Leu/–His/–Ade+X-α-Gal/Aba medium at 30°C for 3 days.

### Metabolomic comparison via UPLC-MS.

The WT and mutant strains were inoculated into PD broth at 28°C and 180 rpm for 5 days, and then the fermentation broth was extracted using ethyl acetate and dried under a vacuum. The dried crude extract samples were dissolved into methanol, and the volume was fixed to 1 mL according to the dry weight of mycelia of each sample and the mass volume ratio. The experiment was repeated three times independently and analyzed by LC-MS using a Thermo Scientific Dionex Ultimate 3000 UHPLC system with a Thermo high-resolution Q Exactive focus mass spectrometer (Thermo, Bremen, Germany). Untargeted metabolomics analysis was performed using Compounds Discoverer 3.0 software (Thermo Fisher Scientific, Carlsbad, CA).

### Statistical analysis.

All experiments were repeated three times, and the data are expressed as the means ± the standard deviations (SD). Prism 8.0 (GraphPad Software, San Diego, CA) was used as the tool for statistical analysis, with one-way analysis of variance, followed by Tukey’s honestly significant difference (HSD) test performed with a set of *P < *0.05.

### Data availability.

All data generated or analyzed during this study are included here or in the associated supplemental material. RNA-Seq and DAP-Seq data were deposited in the NCBI Gene Expression Omnibus (GEO) database and are accessible under GEO series accession numbers GSE213447 (RNA-Seq) and GSE213449 (DAP-Seq).
