# Supplementary material for: AoSte12 Is Required for Mycelial Development, Conidiation, Trap Morphogenesis, and Secondary Metabolism by Regulating Hyphal Fusion in Nematode-Trapping Fungus Arthrobotrys oligospora
Source: Microbiol Spectr. 2023 Feb 14;11(2):e03957-22. doi: 10.1128/spectrum.03957-22 (PMC10101105; doi:10.1128/spectrum.03957-22)
Supplement: Supplemental file 1 — Supplemental material. Download spectrum.03957-22-s0001.pdf, PDF file, 1.7 MB [file spectrum.03957-22-s0001.pdf]

SUPPLEMENTAL MATERIAL

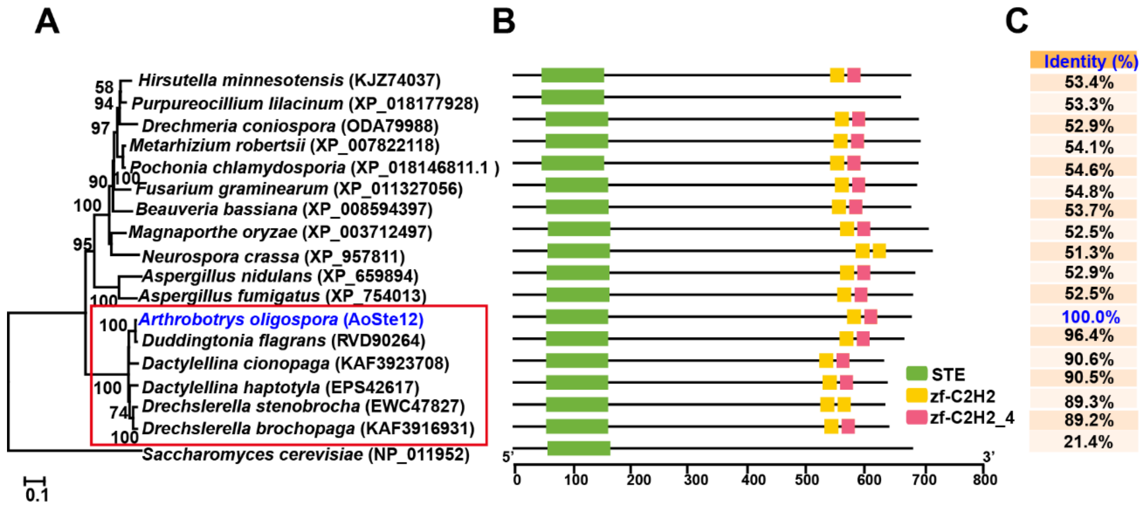

Fig. S1 Phylogenetic analysis, conserved domain, and sequence similarity comparison of Ste12 homologues from different fungi. (A) Phylogenetic tree of Ste12 orthologs from diverse fungi. Phylogenetic tree was constructed using MEGA7.0 software; *A. oligospora* is shown in blue. The nematode-trapping fungi were highlighted with a red frame. (B) Conserved domain of Ste12 homologous proteins. The structural domains of these sequences were analyzed using the Pfam website (http://pfam.xfam.org/). (C) Comparison of sequence similarity between AoSte12 and other homologous proteins. The sequence similarity between AoSte12 and other homologous proteins was analyzed using DNAMAN software.

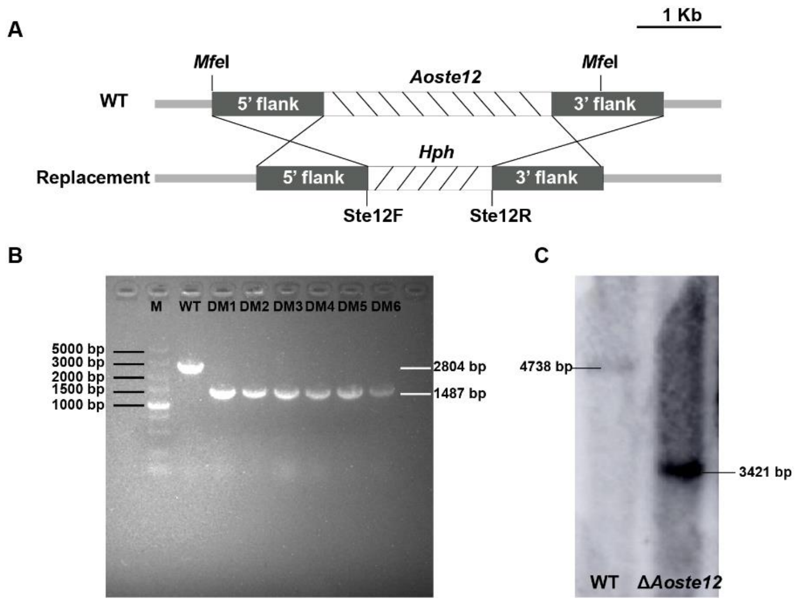

Fig. S2 *Aoste12* disruption and verification of transformants. (A) The homologous

recombination pattern of *Aoste12*. Restriction enzyme cutting site is *Mfe*I. Bar = 1 kb. (B) *Aoste12* disruption transformants were confirmed via PCR amplification. M, DNA marker. WT, wild-type strain. DM1-DM6, Δ*Aoste12* transformants. (C) Southern blotting analysis of wild-type strain (WT) and Δ*Aoste12* transformants.

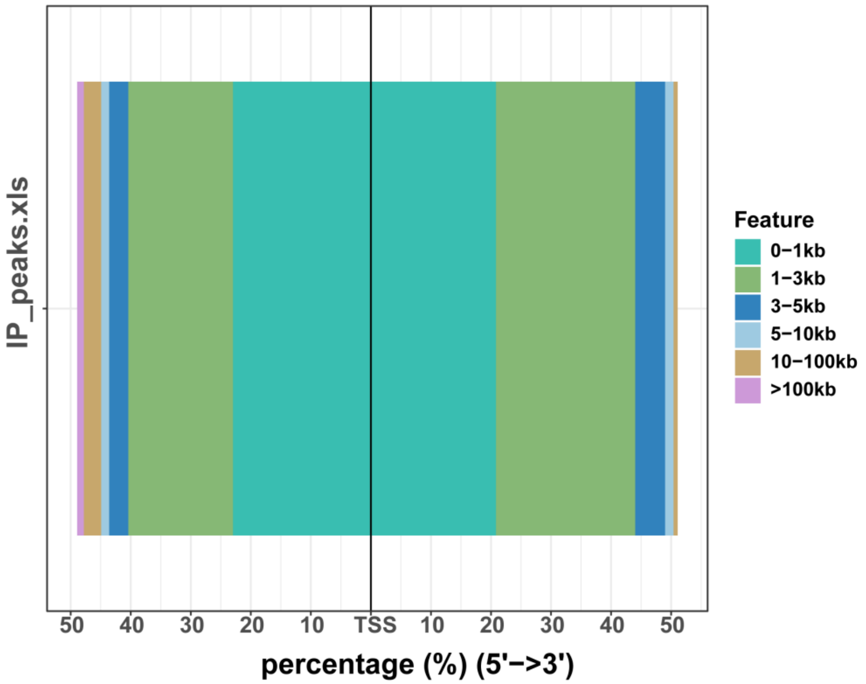

Fig. S3 Distribution ratio of peak to transcriptional start site (TSS) distance of each sample.

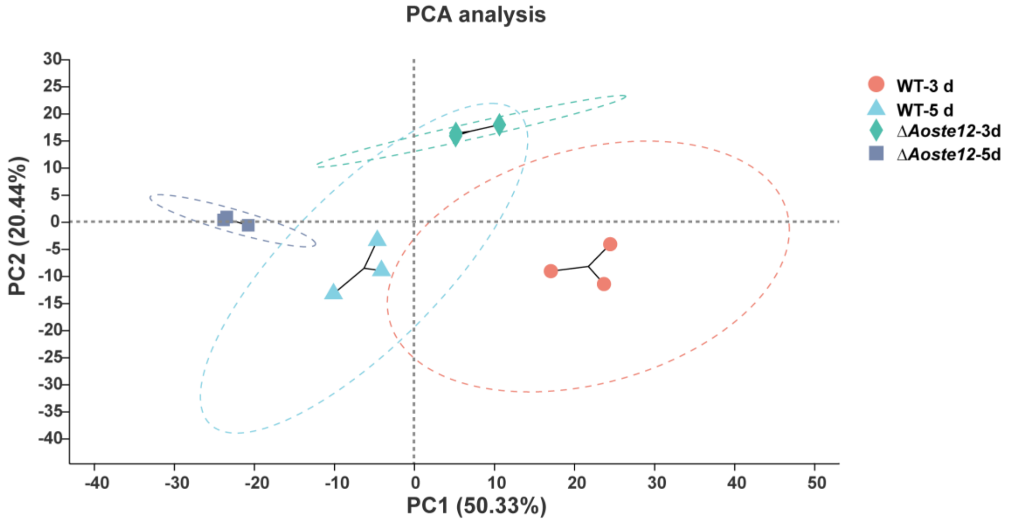

Fig. S4 Principal component analysis (PCA). PCA analysis between the wild-type (WT)

and Δ*Aoste12* mutant samples in the transcriptome.

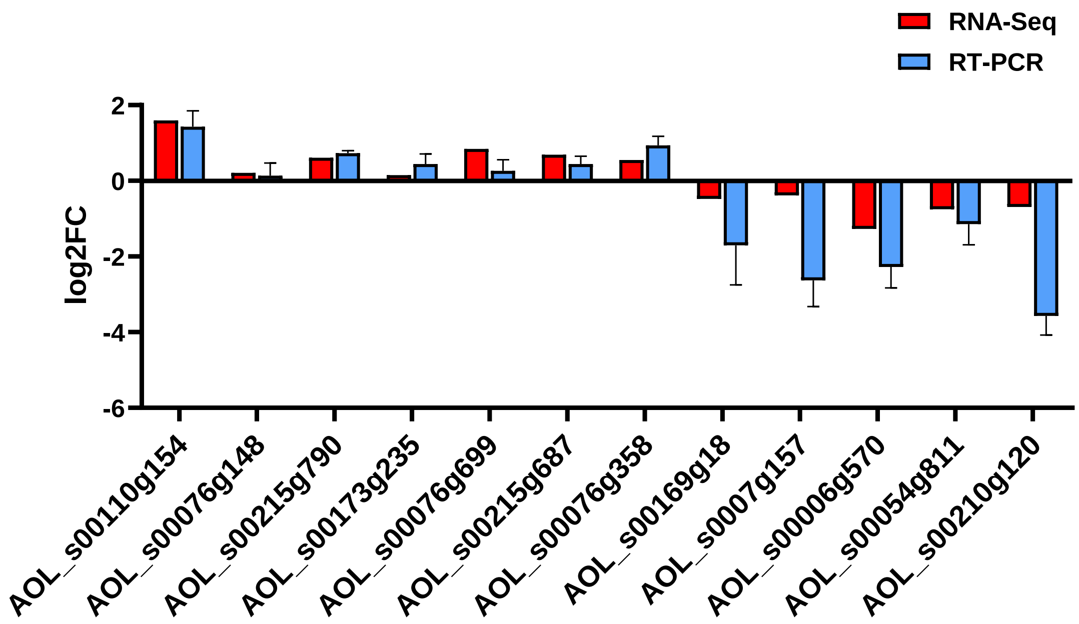

Fig. S5 Verification of transcriptome data using real-time quantitative polymerase chain reaction (RT-qPCR) analysis.

Table S1 List of primers used in this study.

Primers for gene disruption		
Primers	Sequence (5'-3')	Description
Ste12LF1	GTAACGCCAGGGTTTTCCCAGTCACGACGAAGCTGTTGTCTGTCT CGGG	Amplify the *Ste12* gene 5' flank
Ste12LR1	ATCCACTTAACGTTACTGAAATCTCCAACGTGTGTGAAAGTGTGCG ACC	
Ste12RF2	CTCCTTCAATATCATCTTCTGTCTCCGACGTACTTTGGGATGCGGC TTG	Amplify the *Ste12* gene 3' flank
Ste12RR2	GCGGATAACAATTTCACACAGGAAACAGCGCTATCCACCGTTACCC CTC	
hphF	GTCGGAGACAGAAGATGATATTGAAGGAGC	Amplify the hph cassette
hphR	GTTGGAGATTTCAGTAACGTTAAGTGGAT	
Ste12F	GGTCGCACACTTTCACACAC	Verify the transformants
Ste12R	CAAGCCGCATCCCAAAGTAC	
Ste12TZF	CTCGGGCGCTCTTCTACTTT	Make Southern blotting probe
Ste12TZR	GTACCCAAGGAGCGATGTGA	

Primers for real-time quantitative polymerase chain reaction (RT-qPCR) analysis			
Genes	Primers (5'-3')	Genes	Primers (5'-3')

AOL_s00210g120 (*medA*)	120F-TCCGGCCCAATGATTCAGAA<br>120R-AGATCGCAGGAACATGGTGA	AOL_s00054g811 (*velB*)	811F-ATTCCGCAACTTCTCCCTCA<br>811R-GGCATGTTTGGATTCTGGGG
AOL_s00007g157 (*flbC*)	157F-CTCTCCGGCAAAGACAATCG<br>157R-GTCGACTGAGGATAGTAGCT	AOL_s00215g516 (*flbA*)	516F-TTCAAACGCAGCTCCTTCAC<br>516R-TTCAAACGCAGCTCCTTCAC
AOL_s00080g93 (*lreB*)	93F-CCAGGGTCGTCAGTATCTT<br>93R-CAGCATCTTCCAGGTCAA	AOL_s00083g25 (*stuA*)	25F-AGCTCCCGAAACGAGTCTAA<br>25R-ATTGATCATGTGATTATCCT
AOL_s00169g18 (*veA*)	18F-AAGCTACACCCAATCAACGC<br>18R-TTGCGATGCTGACGATCTTG	AOL_s00080g63 (*abaA*)	63F-AACTTTATGCGCCTTGTCGT<br>63R-TTGGCTAGGTGGTCTGTACG
AOL_s00083g487 (*lreA*)	487F-TTCTCTTCGTCCCAAGCCAC<br>487R-ACCGGTTCGAGTGGAGTCTA	AOL_s00043g361 (*fluG*)	361F-GATTCCAGTCCCGTGAATTC<br>361R-GCTAAGGAGAGGATGGGCAT
AOL_s00173g221 (*wetA*)	221F-TTACATGCCACCCCAAGTCC<br>221R-CAATTGCAACTGCGTCCACA	AOL_s00006g570 (*hyp1*)	570F-GCGGATCCAACATGAAGCTT<br>570R-GGTTGACAACTGGGATGCTG
AOL_s00075g211 (*nsdD*)	211F-ATTACGGCCGCCTAGTAGTC<br>211R-CTCGTTTGGACCTGGTTGTG	AOL_s00097g514 (*brlA*)	514F-TTGAGGCCTCGATCCGTAGA<br>514R-AGGTAGATGGCGCTGTTACG
AOL_s00054g700 (*vosA*)	700F-CAAACCACCCACCACCAAAT<br>700R-GGATGGACAGGAGAAGGAC<br>C	AOL_s00076g640 (β-tubulin gene)	640F-CCACCTTCGTCGGTAACTC<br>640R-TCGTCCATACCCTCACCAG
AOL_s00110g154 (*fus3*)	154F-GGACCTCTCAGACGACCATT<br>G<br>154R-AGCACGGTACCATCTGGTTG	AOL_s00076g148	148F-TGTTGTGGTGCAGGTCTTGT<br>148R-ATCCGTGCCAAAGAGCTCAA
AOL_s00215g790	790F-CCCCTAAGCGCTCTCAACAA<br>790R-GGAGATGTGTTCTTTGCGGC	AOL_s00173g235 (*slt2*)	235F-ACAGAGATCTCAAGCCCGGA<br>235R-GCCAGAATACATCCGACCGA
AOL_s00076g699 (*mkk2*)	699F-AACAGATCCAAACCCAGCGA<br>699R-CCTCGGCGATTTTCCCTAGA	AOL_s00215g687 (*ste11*)	687F-ATCCATCGCGACATCAAGGG<br>687R-GGTCTCCCGTGAACATCTCC
AOL_s00076g358 (*far11*)	358F-TCGAACTTGACGGTGTTGCT<br>358R-TGTCAGATATCAGCGCGACC		

Primers for vector construction in yeast two-hybrid analysis

Primers	Sequence (5'-3')
BD-AOL_s00007g203 F	TGCATATGGCCATGGAGGCCATGCCGAACCATCGCCCATC
BD-AOL_s00007g203 R	GCAGGTCGACGGATCCCCGGTAAGTTTTCTGTAGAGTCTT
BD-AOL_s00215g688 F	TGCATATGGCCATGGAGGCCATGGCGTTCAACCCAGACGA
BD-AOL_s00215g688 R	GCAGGTCGACGGATCCCCGGAACAGCCCACTCGCTCTTAT
BD-AOL_s00215g705 F	TGCATATGGCCATGGAGGCCATGTCTTCCTCAAACGAAAA
BD-AOL_s00215g705 R	GCAGGTCGACGGATCCCCGGGTTCGTCTTCCCCAGTCCTT
BD-AOL_s00215g489 F	TGCATATGGCCATGGAGGCCATGGACAATATTCGCGAGGA
BD-AOL_s00215g489 R	GCAGGTCGACGGATCCCCGGGCTTTCAATATCAACCATGA
BD-AOL_s00054g134 F	TGCATATGGCCATGGAGGCCATGGCAGCTCCGACGACTGT
BD-AOL_s00054g134 R	GCAGGTCGACGGATCCCCGGTTTAGCGCCCCATAGTCCTT
BD-AOL_s00110g154 F	TGCATATGGCCATGGAGGCCATGTCTCGCCAGAACTCGAG
BD-AOL_s00110g154 R	GCAGGTCGACGGATCCCCGGCCGCATGATTTCTTCGAAGA
AD-AOL_s00079g294 F	ATATGGCCATGGAGGCCAGTATGTATTCACACAACGCCCT
AD-AOL_s00079g294 R	GCAGGTCGACGGATCCCCGGCATCTGGGGTATCAGTTGCG

Table S2 Statistical sequencing data of transcriptome.

Sample	Total reads	Total mapped	Multiple mapped	Uniquely mapped	Raw reads
WT_5_1	46642548	45155026(96.81%)	174999(0.38%)	44980027(96.44%)	46987920
WT_5_2	48016092	46468497(96.78%)	168177(0.35%)	46300320(96.43%)	48415728
WT_5_3	44695480	43206852(96.67%)	196719(0.44%)	43010133(96.23%)	45068496
WT_7_1	43716240	42292286(96.74%)	165975(0.38%)	42126311(96.36%)	44088204
WT_7_2	42222520	40782732(96.59%)	145438(0.34%)	40637294(96.25%)	42614272
WT_7_3	42706532	41220257(96.52%)	142077(0.33%)	41078180(96.19%)	43132588
Δ*Aoste12*_5_1	42543314	41149610(96.72%)	159095(0.37%)	40990515(96.35%)	42845232
Δ*Aoste12*_5_2	42283656	40918775(96.77%)	152686(0.36%)	40766089(96.41%)	42612150
Δ*Aoste12*_5_3	40777528	39436684(96.71%)	156251(0.38%)	39280433(96.33%)	41128590
Δ*Aoste12*_7_1	42072704	40680645(96.69%)	147780(0.35%)	40532865(96.34%)	42464108
Δ*Aoste12*_7_2	45055222	43517034(96.59%)	136581(0.3%)	43380453(96.28%)	45410612
Δ*Aoste12*_7_3	41361094	40065821(96.87%)	112608(0.27%)	39953213(96.6%)	41703496
**Clean reads**	**Clean bases**	**Error rate(%)**	**Q20(%)**	**Q30(%)**	**GC content(%)**
46642548	6941988390	0.0251	97.99	94.04	48.43
48016092	7142904635	0.0252	97.93	93.92	48.29
44695480	6659282335	0.0254	97.87	93.78	48.31
43716240	6512413572	0.0251	98	94.08	48.21
42222520	6281450334	0.0252	97.93	93.96	48.21
42706532	6362163100	0.0255	97.81	93.65	48.15
42543314	6336524768	0.0249	98.06	94.21	47.82
42283656	6294217582	0.0252	97.97	93.99	47.94
40777528	6067336113	0.0253	97.9	93.86	47.9
42072704	6261458043	0.0257	97.75	93.49	48.21
45055222	6701074624	0.0252	97.95	93.98	48.19
41361094	6176791513	0.0252	97.94	93.94	48.36

Table S3 The interacting protein network nodes prediction of AoSte12 by String.

#node1	node2	node1_string_id	node2_string_id	neighborhood_on_chromosome	gene_fusion	phylogenetic_cooccurrence	homology	coexpression	experimentally_determined_interaction	database_annotated	automated_textmining	combined_score
G1WY29	G1WZ64	756982.G1WY29	756982.G1WZ64	0	0	0	0	0	0	0	0.411	0.411
G1WY29	G1X2X4	756982.G1	756982.G1	0	0	0	0	0	0	0	0.458	0.458

		WY29	X2X4									
G1WY29	G1X591	756982.G1WY29	756982.G1X591	0	0	0	0	0	0	0	0.406	0.406
G1WY29	G1X8N2	756982.G1WY29	756982.G1X8N2	0	0		0	0	0	0	0.567	0.567
G1WY29	G1XAJ0	756982.G1WY29	756982.G1XAJ0	0	0	0	0	0	0.152	0	0.4	0.469
G1WY29	G1XG07	756982.G1WY29	756982.G1XG07	0	0	0	0	0	0.228	0.82	0.428	0.914
G1WY29	G1XJZ4	756982.G1WY29	756982.G1XJZ4	0	0	0	0	0	0.23	0	0.368	0.493
G1WY29	G1XLU8	756982.G1WY29	756982.G1XLU8	0	0	0	0	0	0	0	0.448	0.448
G1WYK2	G1X4I9	756982.G1WYK2	756982.G1X4I9	0	0	0	0	0.085	0	0.816	0.497	0.908
G1WYK2	G1X591	756982.G1WYK2	756982.G1X591	0	0	0	0	0	0	0	0.65	0.65
G1WYK2	G1X7V0	756982.G1WYK2	756982.G1X7V0	0	0	0	0	0	0.507	0	0	0.507
G1WYK2	G1XKT9	756982.G1WYK2	756982.G1XKT9	0.063	0	0	0	0.213	0	0	0.336	0.468
G1WYK2	G1XN30	756982.G1WYK2	756982.G1XN30	0.06	0	0	0	0.078	0	0	0.406	0.44
G1WYK2	G1XNK0	756982.G1WYK2	756982.G1XNK0	0.047	0	0	0	0.115	0	0.993	0.235	0.995
G1WYK2	G1XNK1	756982.G1WYK2	756982.G1XNK1	0.047	0	0	0	0.115	0	0.993	0.235	0.995
G1WYK2	G1XT67	756982.G1WYK2	756982.G1XT67	0.061	0	0	0	0.516	0	0.993	0.689	0.999
G1WYK2	G1XTA1	756982.G1WYK2	756982.G1XTA1	0.063	0	0	0	0	0	0.394	0.103	0.446
G1WYK2	G1XV10	756982.G1WYK2	756982.G1XV10	0	0	0	0	0	0	0	0.428	0.428
G1WYK7	G1WZZ5	756982.G1WYK7	756982.G1WZZ5	0	0	0	0	0	0.265	0	0.266	0.437
G1WYK7	G1X0E4	756982.G1WYK7	756982.G1X0E4	0	0	0	0	0.273	0.268	0.893	0.578	0.973
G1WYK7	G1X1H4	756982.G1WYK7	756982.G1X1H4	0	0	0	0	0.182	0.839	0.893	0.982	0.999
G1WYK7	G1X4S9	756982.G1WYK7	756982.G1X4S9	0	0	0	0	0.139	0.418	0	0.132	0.527
G1WYK7	G1X6C3	756982.G1	756982.G1	0	0	0	0	0	0	0.744	0.262	0.803

		WYK7	X6C3									
G1WYK7	MCM7	756982.G1WYK7	756982.G1X7C5	0	0	0	0	0.143	0	0	0.391	0.456
G1WYK7	G1XE09	756982.G1WYK7	756982.G1XE09	0	0	0	0	0.183	0	0.601	0.881	0.958
G1WYK7	G1XFS2	756982.G1WYK7	756982.G1XFS2	0	0	0	0	0.186	0	0.893	0.512	0.954
G1WYK7	G1XGS2	756982.G1WYK7	756982.G1XGS2	0	0	0	0	0.284	0.518	0	0.248	0.718
G1WYK7	G1XLV9	756982.G1WYK7	756982.G1XLV9	0	0	0	0	0	0.587	0	0	0.587
G1WYQ0	G1X286	756982.G1WYQ0	756982.G1X286	0	0	0	0	0	0.481	0	0.133	0.531
G1WYQ0	G1X496	756982.G1WYQ0	756982.G1X496	0	0	0	0	0.502	0.688	0	0.352	0.891
G1WYQ0	G1X645	756982.G1WYQ0	756982.G1X645	0	0	0	0	0.255	0	0	0.344	0.49
G1WYQ0	G1X7V0	756982.G1WYQ0	756982.G1X7V0	0	0	0	0	0.355	0.495	0	0	0.66
G1WYQ0	G1XB71	756982.G1WYQ0	756982.G1XB71	0	0	0	0	0.406	0.672	0.993	0.779	0.999
G1WYQ0	G1XCI8	756982.G1WYQ0	756982.G1XCI8	0	0	0	0	0.195	0.518	0	0	0.595
G1WYQ0	G1XLK0	756982.G1WYQ0	756982.G1XLK0	0	0	0	0	0	0.508	0	0	0.508
G1WYS7	G1X2E4	756982.G1WYS7	756982.G1X2E4	0	0	0	0	0	0.936	0	0.142	0.943
G1WYS7	G1X2N7	756982.G1WYS7	756982.G1X2N7	0	0	0	0	0	0	0.502	0.173	0.571
G1WYS7	G1X6W7	756982.G1WYS7	756982.G1X6W7	0	0	0	0	0.142	0.152	0.601	0	0.684
G1WYS7	G1X7E7	756982.G1WYS7	756982.G1X7E7	0	0	0	0.374	0	0	0.385	0.097	0.407
G1WYS7	G1XBE9	756982.G1WYS7	756982.G1XBE9	0	0	0	0	0	0.456	0	0.128	0.505
G1WYS7	G1XCX4	756982.G1WYS7	756982.G1XCX4	0	0	0	0	0.459	0	0	0	0.459
G1WYS7	G1XJZ4	756982.G1WYS7	756982.G1XJZ4	0	0	0	0	0	0.611	0.492	0.415	0.874
G1WYS7	G1XKY4	756982.G1WYS7	756982.G1XKY4	0	0	0	0	0.222	0.692	0.492	0.775	0.969
G1WYS7	G1XLU8	756982.G1	756982.G1	0	0	0	0	0	0.505	0	0.258	0.617

		WYS7	XLU8									
G1WYS7	G1XRF2	756982.G1WYS7	756982.G1XRF2	0	0	0	0	0.345	0	0	0.361	0.564
G1WZ64	G1WZY0	756982.G1WZ64	756982.G1WZY0	0	0	0	0	0.235	0.495	0	0.444	0.766
G1WZ64	G1X344	756982.G1WZ64	756982.G1X344	0	0	0	0	0.355	0.15	0	0.41	0.648
G1WZ64	G1X370	756982.G1WZ64	756982.G1X370	0	0	0	0	0	0.503	0	0.411	0.695
G1WZ64	G1X4I9	756982.G1WZ64	756982.G1X4I9	0	0	0	0	0.436	0	0	0.398	0.646
G1WZ64	G1X8N2	756982.G1WZ64	756982.G1X8N2	0	0	0	0	0	0	0	0.654	0.654
G1WZ64	G1XAJ0	756982.G1WZ64	756982.G1XAJ0	0	0	0	0	0.519	0.699	0.993	0.961	0.999
G1WZ64	G1XFG1	756982.G1WZ64	756982.G1XFG1	0	0	0	0	0	0	0.856	0	0.856
G1WZ64	G1XFQ1	756982.G1WZ64	756982.G1XFQ1	0	0	0	0	0	0	0	0.431	0.431
G1WZ64	G1XI99	756982.G1WZ64	756982.G1XI99	0	0	0	0	0	0	0.989	0	0.989
G1WZ64	G1XIF7	756982.G1WZ64	756982.G1XIF7	0	0	0	0	0	0.482	0	0	0.482
G1WZ64	G1XKT9	756982.G1WZ64	756982.G1XKT9	0	0	0	0	0.209	0	0	0.365	0.476
G1WZ64	G1XLK2	756982.G1WZ64	756982.G1XLK2	0	0	0	0	0	0.505	0.624	0.431	0.885
G1WZ64	G1XSV0	756982.G1WZ64	756982.G1XSV0	0	0	0	0	0	0.505	0	0.446	0.714
G1WZ64	G1XTA1	756982.G1WZ64	756982.G1XTA1	0	0	0	0	0	0	0.993	0	0.993
G1WZ64	G1XV29	756982.G1WZ64	756982.G1XV29	0	0	0	0	0	0.225	0	0.642	0.711
G1WZI7	G1X496	756982.G1WZI7	756982.G1X496	0	0	0	0.376	0.186	0.505	0	0.88	0.809
G1WZI7	G1X7V0	756982.G1WZI7	756982.G1X7V0	0	0	0	0	0	0	0	0.507	0.507
G1WZQ4	G1X2X4	756982.G1WZQ4	756982.G1X2X4	0	0	0	0	0	0.609	0	0	0.609
G1WZQ4	G1X8N6	756982.G1WZQ4	756982.G1X8N6	0	0	0	0	0	0.222	0	0.606	0.68
G1WZQ4	G1XJZ4	756982.G1	756982.G1	0	0	0	0.29	0	0.505	0	0.401	0.637

		WZQ4	XJZ4									
G1WZQ4	G1XLU8	756982.G1WZQ4	756982.G1XLU8	0	0	0	0	0	0.526	0	0.243	0.626
G1WZV7	G1X4H2	756982.G1WZV7	756982.G1X4H2	0	0	0	0	0.183	0.607	0	0.262	0.742
G1WZV7	G1X645	756982.G1WZV7	756982.G1X645	0	0	0	0	0.195	0	0	0.634	0.693
G1WZV7	G1XGS2	756982.G1WZV7	756982.G1XGS2	0	0	0	0	0.198	0	0	0.491	0.574
G1WZY0	G1X2E4	756982.G1WZY0	756982.G1X2E4	0.461	0	0	0	0.106	0	0	0.259	0.612
G1WZY0	G1X4K4	756982.G1WZY0	756982.G1X4K4	0	0	0	0	0.441	0.533	0	0.235	0.783
G1WZY0	G1X4S9	756982.G1WZY0	756982.G1X4S9	0.126	0	0	0	0.111	0	0	0.817	0.845
G1WZY0	G1X7V7	756982.G1WZY0	756982.G1X7V7	0.131	0	0	0	0	0	0.816	0.232	0.866
G1WZY0	G1X8N2	756982.G1WZY0	756982.G1X8N2	0	0	0	0	0.124	0	0	0.411	0.462
G1WZY0	G1XAJ0	756982.G1WZY0	756982.G1XAJ0	0	0	0	0	0.278	0.431	0	0.549	0.799
G1WZY0	G1XBB2	756982.G1WZY0	756982.G1XBB2	0.081	0	0	0	0.28	0	0	0.345	0.529
G1WZY0	G1XD36	756982.G1WZY0	756982.G1XD36	0.065	0	0	0	0	0.225	0	0.726	0.784
G1WZY0	G1XI36	756982.G1WZY0	756982.G1XI36	0.076	0	0	0	0	0	0	0.382	0.405
G1WZY0	G1XKT9	756982.G1WZY0	756982.G1XKT9	0.112	0	0	0	0.523	0.495	0.993	0.78	0.999
G1WZY0	G1XLU8	756982.G1WZY0	756982.G1XLU8	0	0	0	0	0	0	0	0.475	0.475
G1WZY0	G1XLV4	756982.G1WZY0	756982.G1XLV4	0	0	0	0	0.103	0.356	0	0.095	0.432
G1WZY0	G1XNH2	756982.G1WZY0	756982.G1XNH2	0	0	0	0	0	0	0	0.427	0.427
G1WZY0	G1XTC2	756982.G1WZY0	756982.G1XTC2	0	0	0	0	0.449	0	0	0.347	0.625
G1WZY0	G1XTD6	756982.G1WZY0	756982.G1XTD6	0.09	0	0	0	0	0	0	0.541	0.564
G1WZY0	G1XUH5	756982.G1WZY0	756982.G1XUH5	0	0	0	0	0	0.5	0	0.26	0.614
G1WZZ5	G1X0E4	756982.G1	756982.G1	0	0	0	0	0	0.236	0.599	0.153	0.718

		WZZ5	X0E4									
G1WZZ5	G1X4S9	756982.G1WZZ5	756982.G1X4S9	0	0	0	0	0	0.621	0.599	0.314	0.887
G1WZZ5	G1XER5	756982.G1WZZ5	756982.G1XER5	0	0	0	0	0.105	0.259	0	0.858	0.898
G1WZZ5	G1XFS2	756982.G1WZZ5	756982.G1XFS2	0	0	0	0	0.138	0	0.599	0.119	0.669
G1X070	G1XBB2	756982.G1X070	756982.G1XBB2	0	0	0	0	0.355	0	0	0.292	0.524
G1X070	G1XV10	756982.G1X070	756982.G1XV10	0	0	0	0	0	0	0.718	0.262	0.783
G1X0A0	G1X0E4	756982.G1X0A0	756982.G1X0E4	0	0	0	0	0.626	0.275	0.917	0.481	0.987
G1X0A0	G1X1H4	756982.G1X0A0	756982.G1X1H4	0	0	0	0	0.158	0.544	0	0.368	0.736
G1X0A0	G1X286	756982.G1X0A0	756982.G1X286	0	0	0	0.469	0.915	0.998	0.972	0.96	0.999
G1X0A0	G1X4S9	756982.G1X0A0	756982.G1X4S9	0	0	0	0	0.931	0	0	0.795	0.985
G1X0A0	G1X4X7	756982.G1X0A0	756982.G1X4X7	0	0	0	0	0.679	0.968	0.946	0.938	0.999
G1X0A0	G1X6C3	756982.G1X0A0	756982.G1X6C3	0	0	0	0	0	0	0.744	0	0.744
G1X0A0	G1X6W7	756982.G1X0A0	756982.G1X6W7	0	0	0.216	0	0.202	0	0	0.174	0.438
G1X0A0	MCM7	756982.G1X0A0	756982.G1X7C5	0	0	0	0.479	0.914	0.998	0.599	0.96	0.999
G1X0A0	G1XE09	756982.G1X0A0	756982.G1XE09	0	0	0	0	0.408	0.275	0.6	0.473	0.897
G1X0A0	G1XFS2	756982.G1X0A0	756982.G1XFS2	0	0	0.209	0	0.853	0.514	0	0.722	0.982
G1X0A0	G1XG07	756982.G1X0A0	756982.G1XG07	0	0	0	0	0.402	0	0	0	0.402
G1X0A0	G1XGS2	756982.G1X0A0	756982.G1XGS2	0	0	0	0	0.654	0	0.599	0.326	0.898
G1X0A0	G1XHC4	756982.G1X0A0	756982.G1XHC4	0	0	0	0	0.3	0.259	0	0.13	0.509
G1X0A0	G1XIF7	756982.G1X0A0	756982.G1XIF7	0	0	0.263	0	0.239	0	0	0	0.415
G1X0A0	G1XLU8	756982.G1X0A0	756982.G1XLU8	0	0	0	0	0	0.472	0	0.443	0.693
G1X0A0	G1XLV4	756982.G1	756982.G1	0	0	0	0	0.10	0.628	0.702	0.173	0.907

		X0A0	XLV4					3				
G1X0A0	G1XT19	756982.G1 X0A0	756982.G1 XT19	0	0	0	0	0.59 6	0.869	0.993	0.906	0.999
G1X0B0	G1X4I9	756982.G1 X0B0	756982.G1 X4I9	0	0	0	0	0	0.495	0	0.168	0.562
G1X0B0	G1X4K4	756982.G1 X0B0	756982.G1 X4K4	0	0	0	0	0.34 9	0	0	0.978	0.985
G1X0B0	G1X7E7	756982.G1 X0B0	756982.G1 X7E7	0	0	0	0	0.38 9	0	0	0.261	0.529
G1X0B0	G1X8L2	756982.G1 X0B0	756982.G1 X8L2	0	0	0	0	0	0	0.4	0.095	0.434
G1X0B0	G1XJL2	756982.G1 X0B0	756982.G1 XJL2	0	0	0	0	0.24	0	0	0.72	0.778
G1X0E4	G1X1H4	756982.G1 X0E4	756982.G1 X1H4	0	0	0	0	0.14 3	0.639	0.918	0.693	0.991
G1X0E4	G1X286	756982.G1 X0E4	756982.G1 X286	0	0	0	0	0.22 2	0.227	0.802	0.426	0.923
G1X0E4	G1X4S9	756982.G1 X0E4	756982.G1 X4S9	0	0	0	0	0.94 6	0.275	0.893	0.653	0.998
G1X0E4	G1X4X7	756982.G1 X0E4	756982.G1 X4X7	0	0	0	0	0.62	0.415	0	0.31	0.833
G1X0E4	G1X6C3	756982.G1 X0E4	756982.G1 X6C3	0	0	0	0	0	0	0.993	0.845	0.999
G1X0E4	MCM7	756982.G1 X0E4	756982.G1 X7C5	0	0	0	0	0.70 5	0.378	0.917	0.581	0.993
G1X0E4	G1XE09	756982.G1 X0E4	756982.G1 XE09	0	0	0	0	0.32 9	0.238	0.606	0.674	0.926
G1X0E4	G1XER5	756982.G1 X0E4	756982.G1 XER5	0	0	0	0	0.11 5	0	0.893	0.17	0.915
G1X0E4	G1XFS2	756982.G1 X0E4	756982.G1 XFS2	0	0	0	0	0.60 1	0.565	0.923	0.411	0.991
G1X0E4	G1XG07	756982.G1 X0E4	756982.G1 XG07	0	0	0	0	0.42 5	0	0	0	0.425
G1X0E4	G1XGS2	756982.G1 X0E4	756982.G1 XGS2	0	0	0	0	0.36 6	0.436	0.6	0.265	0.881
G1X0E4	G1XIG5	756982.G1 X0E4	756982.G1 XIG5	0	0	0	0	0.11 5	0	0	0.392	0.439
G1X0E4	G1XLV4	756982.G1 X0E4	756982.G1 XLV4	0	0	0	0	0.10 6	0.577	0	0.097	0.629
G1X0E4	G1XLV9	756982.G1 X0E4	756982.G1 XLV9	0	0	0	0	0	0	0	0.422	0.422
G1X0E4	G1XMH7	756982.G1	756982.G1	0	0	0	0	0.28	0.196	0	0.415	0.635

		X0E4	XMH7					7				
G1X0E4	G1XT19	756982.G1X0E4	756982.G1XT19	0	0	0	0	0.188	0.225	0.802	0.13	0.877
G1X1H4	G1X286	756982.G1X1H4	756982.G1X286	0	0	0	0	0.391	0.533	0	0.325	0.791
G1X1H4	G1X4S9	756982.G1X1H4	756982.G1X4S9	0.063	0	0	0	0.143	0.631	0	0.165	0.719
G1X1H4	G1X4X7	756982.G1X1H4	756982.G1X4X7	0	0	0	0	0	0.545	0	0.299	0.667
G1X1H4	G1X6C3	756982.G1X1H4	756982.G1X6C3	0	0	0	0	0	0.224	0.744	0.261	0.84
G1X1H4	MCM7	756982.G1X1H4	756982.G1X7C5	0	0	0	0	0.183	0.621	0.599	0.764	0.967
G1X1H4	G1X8N3	756982.G1X1H4	756982.G1X8N3	0	0	0	0	0.197	0	0	0.347	0.453
G1X1H4	G1XE09	756982.G1X1H4	756982.G1XE09	0	0	0	0	0.278	0.648	0.601	0.945	0.994
G1X1H4	G1XER5	756982.G1X1H4	756982.G1XER5	0	0	0	0	0.128	0	0	0.468	0.516
G1X1H4	G1XFS2	756982.G1X1H4	756982.G1XFS2	0	0	0	0.156	0.191	0.941	0.955	0.815	0.999
G1X1H4	G1XGS2	756982.G1X1H4	756982.G1XGS2	0	0	0	0	0.307	0.58	0	0.386	0.806
G1X1H4	G1XHC4	756982.G1X1H4	756982.G1XHC4	0	0	0	0	0.416	0	0	0	0.416
G1X1H4	G1XJZ4	756982.G1X1H4	756982.G1XJZ4	0	0	0	0	0	0.505	0	0	0.505
G1X1H4	G1XT19	756982.G1X1H4	756982.G1XT19	0	0	0	0	0.261	0.553	0	0.252	0.731
G1X1Q9	G1X344	756982.G1X1Q9	756982.G1X344	0	0	0	0	0.475	0	0	0	0.475
G1X1Q9	G1X4I9	756982.G1X1Q9	756982.G1X4I9	0	0	0	0	0.353	0	0.621	0.308	0.815
G1X1Q9	G1XNK0	756982.G1X1Q9	756982.G1XNK0	0	0	0	0	0.349	0	0	0.473	0.642
G1X286	G1X4I9	756982.G1X286	756982.G1X4I9	0	0	0	0	0.124	0.431	0	0.283	0.611
G1X286	G1X4S9	756982.G1X286	756982.G1X4S9	0	0	0	0	0.923	0	0	0.658	0.973
G1X286	G1X4X7	756982.G1X286	756982.G1X4X7	0	0	0	0	0.635	0.978	0.946	0.898	0.999
G1X286	G1X6C3	756982.G1	756982.G1	0	0	0	0	0	0	0.744	0	0.744

		X286	X6C3									
G1X286	G1X6W7	756982.G1X286	756982.G1X6W7	0	0	0.261	0	0.269	0	0	0.212	0.537
G1X286	MCM7	756982.G1X286	756982.G1X7C5	0	0	0	0.498	0.949	0.998	0.972	0.967	0.999
G1X286	G1XB71	756982.G1X286	756982.G1XB71	0	0	0	0	0	0.508	0	0	0.508
G1X286	G1XCI8	756982.G1X286	756982.G1XCI8	0	0	0	0	0.096	0.55	0	0	0.576
G1X286	G1XE09	756982.G1X286	756982.G1XE09	0	0	0	0	0.42	0.275	0.6	0.424	0.89
G1X286	G1XFS2	756982.G1X286	756982.G1XFS2	0	0	0	0	0.861	0.51	0	0.561	0.967
G1X286	G1XGS2	756982.G1X286	756982.G1XGS2	0	0	0	0	0.909	0.498	0.599	0.173	0.983
G1X286	G1XHC4	756982.G1X286	756982.G1XHC4	0	0	0	0	0.402	0	0	0.128	0.456
G1X286	G1XLU8	756982.G1X286	756982.G1XLU8	0	0	0	0	0	0.511	0	0.536	0.763
G1X286	G1XLV4	756982.G1X286	756982.G1XLV4	0	0	0	0	0.104	0.538	0.702	0.218	0.891
G1X286	G1XT19	756982.G1X286	756982.G1XT19	0	0	0	0	0.512	0.736	0.993	0.916	0.999
G1X2E4	G1X4I9	756982.G1X2E4	756982.G1X4I9	0.056	0	0	0	0.13	0	0.608	0.178	0.7
G1X2E4	G1X4S9	756982.G1X2E4	756982.G1X4S9	0.063	0	0	0	0.12	0.227	0	0.421	0.582
G1X2E4	G1X6W7	756982.G1X2E4	756982.G1X6W7	0	0	0	0	0.258	0.206	0	0.089	0.416
G1X2E4	G1X7A4	756982.G1X2E4	756982.G1X7A4	0.461	0	0	0	0.076	0	0	0.627	0.798
G1X2E4	G1XBB2	756982.G1X2E4	756982.G1XBB2	0.084	0	0	0	0.096	0	0	0.405	0.464
G1X2E4	G1XFS2	756982.G1X2E4	756982.G1XFS2	0.06	0	0	0	0.123	0	0.361	0.264	0.56
G1X2E4	G1XGS2	756982.G1X2E4	756982.G1XGS2	0	0	0	0	0.127	0	0	0.383	0.438
G1X2E4	G1XJZ4	756982.G1X2E4	756982.G1XJZ4	0	0	0	0	0	0.304	0.455	0.084	0.622
G1X2E4	G1XKY4	756982.G1X2E4	756982.G1XKY4	0	0	0	0	0	0.304	0.455	0.309	0.715
G1X2E4	G1XLV4	756982.G1	756982.G1	0	0	0	0	0	0.491	0	0.114	0.53

		X2E4	XLV4									
G1X2E4	G1XNK0	756982.G1X2E4	756982.G1XNK0	0.081	0	0	0	0.2	0.388	0	0.345	0.666
G1X2E4	G1XNU1	756982.G1X2E4	756982.G1XNU1	0	0	0	0	0.095	0	0.441	0.135	0.524
G1X2E4	G1XTD6	756982.G1X2E4	756982.G1XTD6	0.112	0	0	0	0.349	0	0	0.127	0.451
G1X2E4	G1XUH5	756982.G1X2E4	756982.G1XUH5	0	0	0	0	0.115	0	0.993	0.127	0.994
G1X2N7	G1X4I9	756982.G1X2N7	756982.G1X4I9	0	0	0	0	0	0.495	0	0.209	0.583
G1X2N7	G1XJZ4	756982.G1X2N7	756982.G1XJZ4	0	0	0	0	0	0.505	0	0.101	0.536
G1X2N7	G1XLK0	756982.G1X2N7	756982.G1XLK0	0	0	0	0	0	0	0.993	0	0.993
G1X2N7	G1XQ38	756982.G1X2N7	756982.G1XQ38	0	0	0	0	0	0.505	0	0	0.505
G1X2X4	G1X591	756982.G1X2X4	756982.G1X591	0	0	0	0	0	0.505	0	0.362	0.671
G1X2X4	G1XJZ4	756982.G1X2X4	756982.G1XJZ4	0	0	0	0	0	0.514	0	0.329	0.66
G1X2X4	G1XKY4	756982.G1X2X4	756982.G1XKY4	0	0	0	0	0	0	0	0.401	0.401
G1X2X4	G1XUH5	756982.G1X2X4	756982.G1XUH5	0	0	0	0	0	0	0.437	0	0.437
G1X344	G1X4I9	756982.G1X344	756982.G1X4I9	0.112	0	0	0	0.571	0.53	0.993	0.366	0.999
G1X344	G1X4K4	756982.G1X344	756982.G1X4K4	0.097	0	0	0	0.205	0	0.478	0.303	0.704
G1X344	G1X8N2	756982.G1X344	756982.G1X8N2	0.063	0	0	0	0.335	0	0	0.539	0.688
G1X344	G1XAJ0	756982.G1X344	756982.G1XAJ0	0	0	0	0	0.326	0	0	0.551	0.684
G1X344	G1XHA0	756982.G1X344	756982.G1XHA0	0	0	0	0	0	0	0.804	0	0.804
G1X344	G1XKT9	756982.G1X344	756982.G1XKT9	0.047	0	0	0	0.354	0	0	0.31	0.538
G1X344	G1XLK2	756982.G1X344	756982.G1XLK2	0	0	0	0	0.236	0	0	0.519	0.617
G1X344	G1XNK0	756982.G1X344	756982.G1XNK0	0.059	0	0	0	0.344	0	0	0.255	0.5
G1X344	G1XNK1	756982.G1	756982.G1	0.059	0	0	0	0.35	0	0	0.459	0.642

		X344	XNK1					4				
G1X344	G1XSV0	756982.G1 X344	756982.G1 XSV0	0	0	0	0	0	0	0	0.574	0.574
G1X344	G1XV29	756982.G1 X344	756982.G1 XV29	0	0	0	0	0.23 9	0	0	0.259	0.412
G1X370	G1X4I9	756982.G1 X370	756982.G1 X4I9	0	0	0	0	0	0	0.498	0	0.498
G1X370	G1X4R4	756982.G1 X370	756982.G1 X4R4	0	0	0	0	0	0	0	0.411	0.411
G1X370	G1X645	756982.G1 X370	756982.G1 X645	0	0	0	0	0.41	0	0	0	0.41
G1X370	G1X8N2	756982.G1 X370	756982.G1 X8N2	0.042	0	0	0	0	0	0	0.419	0.42
G1X370	G1XAJ0	756982.G1 X370	756982.G1 XAJ0	0	0	0	0	0	0.505	0	0.406	0.693
G1X370	G1XBB2	756982.G1 X370	756982.G1 XBB2	0.064	0	0	0	0	0	0.412	0.145	0.488
G1X370	G1XEY1	756982.G1 X370	756982.G1 XEY1	0	0	0	0	0	0	0.456	0	0.456
G1X370	G1XIV2	756982.G1 X370	756982.G1 XIV2	0	0	0	0	0	0	0.718	0.334	0.804
G1X370	G1XKX3	756982.G1 X370	756982.G1 XKX3	0	0	0	0	0	0	0.993	0	0.993
G1X370	G1XKX4	756982.G1 X370	756982.G1 XKX4	0	0	0	0	0.25 7	0	0	0.259	0.426
G1X370	G1XLK2	756982.G1 X370	756982.G1 XLK2	0	0	0	0	0	0	0.869	0.617	0.948
G1X370	G1XQ38	756982.G1 X370	756982.G1 XQ38	0.054	0	0	0	0.23 1	0	0.456	0.105	0.598
G1X370	G1XSV0	756982.G1 X370	756982.G1 XSV0	0	0	0	0	0	0	0	0.431	0.431
G1X370	G1XV29	756982.G1 X370	756982.G1 XV29	0	0	0	0	0	0	0	0.4	0.4
G1X496	G1X645	756982.G1 X496	756982.G1 X645	0	0	0	0	0	0	0	0.41	0.41
G1X496	G1X7V0	756982.G1 X496	756982.G1 X7V0	0	0	0	0	0.25 1	0	0	0.583	0.674
G1X496	G1XB71	756982.G1 X496	756982.G1 XB71	0	0	0	0	0.14	0.204	0	0.208	0.41
G1X4B7	G1X4I9	756982.G1 X4B7	756982.G1 X4I9	0	0	0	0	0.56	0.204	0	0.132	0.669
G1X4B7	G1X5K1	756982.G1	756982.G1	0	0	0	0	0.10	0	0	0.365	0.408

		X4B7	X5K1					6				
G1X4B7	G1X7E7	756982.G1X4B7	756982.G1X7E7	0	0	0	0	0.102	0.279	0	0.216	0.448
G1X4B7	G1X9L3	756982.G1X4B7	756982.G1X9L3	0	0	0	0	0	0	0	0.601	0.601
G1X4B7	G1XLV4	756982.G1X4B7	756982.G1XLV4	0	0	0	0	0	0.534	0	0.222	0.622
G1X4B7	G1XLV9	756982.G1X4B7	756982.G1XLV9	0	0	0	0	0.876	0	0	0	0.876
G1X4B7	G1XR79	756982.G1X4B7	756982.G1XR79	0	0	0	0	0.441	0	0	0	0.441
G1X4B7	G1XTC2	756982.G1X4B7	756982.G1XTC2	0	0	0	0	0.518	0	0	0	0.518
G1X4H2	G1X7V0	756982.G1X4H2	756982.G1X7V0	0	0	0	0	0.198	0.496	0	0.259	0.674
G1X4H2	G1XAL6	756982.G1X4H2	756982.G1XAL6	0	0	0	0	0.389	0.516	0	0.664	0.892
G1X4H2	G1XV70	756982.G1X4H2	756982.G1XV70	0	0	0	0.283	0	0.487	0.6	0.432	0.849
G1X4I9	G1X4K4	756982.G1X4I9	756982.G1X4K4	0.087	0	0	0	0.135	0	0	0.37	0.459
G1X4I9	G1X4X7	756982.G1X4I9	756982.G1X4X7	0	0	0	0	0	0.489	0	0	0.489
G1X4I9	G1X5K5	756982.G1X4I9	756982.G1X5K5	0.059	0	0	0	0	0	0.455	0.118	0.508
G1X4I9	G1X8N2	756982.G1X4I9	756982.G1X8N2	0	0	0	0	0	0.495	0	0.477	0.725
G1X4I9	G1XAJ0	756982.G1X4I9	756982.G1XAJ0	0	0	0	0	0.512	0	0	0.547	0.769
G1X4I9	G1XJL2	756982.G1X4I9	756982.G1XJL2	0	0	0	0	0	0	0.596	0	0.596
G1X4I9	G1XLK2	756982.G1X4I9	756982.G1XLK2	0	0	0	0	0	0	0	0.432	0.432
G1X4I9	G1XNK0	756982.G1X4I9	756982.G1XNK0	0.056	0	0	0	0.703	0	0.993	0.665	0.999
G1X4I9	G1XNK1	756982.G1X4I9	756982.G1XNK1	0.056	0	0	0	0.52	0	0.993	0.433	0.998
G1X4I9	G1XSV0	756982.G1X4I9	756982.G1XSV0	0	0	0	0	0.141	0	0	0.799	0.82
G1X4I9	G1XT67	756982.G1X4I9	756982.G1XT67	0.063	0	0	0	0	0	0.449	0.126	0.509
G1X4I9	G1XV10	756982.G1	756982.G1	0	0	0	0	0	0.485	0	0.371	0.662

		X4I9	XV10									
G1X4K4	G1X5K5	756982.G1X4K4	756982.G1X5K5	0.063	0	0	0	0	0.346	0.993	0.128	0.996
G1X4K4	G1X6W7	756982.G1X4K4	756982.G1X6W7	0	0	0	0	0.205	0	0	0.42	0.519
G1X4K4	G1XFQ1	756982.G1X4K4	756982.G1XFQ1	0	0	0	0	0	0	0	0.41	0.41
G1X4K4	G1XIG5	756982.G1X4K4	756982.G1XIG5	0	0	0	0	0	0.709	0	0	0.709
G1X4K4	G1XJL2	756982.G1X4K4	756982.G1XJL2	0	0	0	0	0.548	0.16	0	0.787	0.912
G1X4K4	G1XJZ4	756982.G1X4K4	756982.G1XJZ4	0	0	0	0	0.198	0	0	0.303	0.417
G1X4K4	G1XMG3	756982.G1X4K4	756982.G1XMG3	0	0	0	0	0.183	0	0	0.391	0.481
G1X4K4	G1XNK0	756982.G1X4K4	756982.G1XNK0	0	0	0	0	0.316	0	0.993	0.363	0.997
G1X4K4	G1XNK1	756982.G1X4K4	756982.G1XNK1	0	0	0	0	0.236	0	0.993	0	0.994
G1X4K4	G1XNU1	756982.G1X4K4	756982.G1XNU1	0	0	0	0	0.139	0	0.441	0.107	0.533
G1X4K4	G1XV10	756982.G1X4K4	756982.G1XV10	0	0	0	0	0.137	0	0.499	0.261	0.653
G1X4R4	G1XLK2	756982.G1X4R4	756982.G1XLK2	0	0	0	0	0	0	0.463	0.471	0.704
G1X4R4	G1XSV0	756982.G1X4R4	756982.G1XSV0	0	0	0	0	0	0	0	0.479	0.479
G1X4R4	G1XV29	756982.G1X4R4	756982.G1XV29	0	0	0	0	0	0.494	0	0.423	0.696
G1X4S9	G1X4X7	756982.G1X4S9	756982.G1X4X7	0	0	0	0	0.52	0	0	0.486	0.743
G1X4S9	G1X6C3	756982.G1X4S9	756982.G1X6C3	0	0	0	0	0	0	0.744	0.257	0.802
G1X4S9	G1X7A4	756982.G1X4S9	756982.G1X7A4	0.461	0	0	0	0	0	0	0	0.461
G1X4S9	MCM7	756982.G1X4S9	756982.G1X7C5	0	0	0	0	0.946	0.275	0	0.714	0.988
G1X4S9	G1XAK8	756982.G1X4S9	756982.G1XAK8	0	0	0	0	0.4	0	0	0	0.4
G1X4S9	G1XE09	756982.G1X4S9	756982.G1XE09	0	0	0	0	0.411	0.759	0	0.334	0.897
G1X4S9	G1XER5	756982.G1	756982.G1	0	0	0	0	0.16	0.932	0.893	0.828	0.999

		X4S9	XER5					9				
G1X4S9	G1XFS2	756982.G1 X4S9	756982.G1 XFS2	0.063	0	0	0	0.39 2	0.871	0.802	0.74	0.996
G1X4S9	G1XG07	756982.G1 X4S9	756982.G1 XG07	0	0	0	0	0.39 3	0	0	0.14	0.456
G1X4S9	G1XGS2	756982.G1 X4S9	756982.G1 XGS2	0.461	0	0	0	0.39 6	0	0.918	0.753	0.993
G1X4S9	G1XHC4	756982.G1 X4S9	756982.G1 XHC4	0	0	0	0	0.52 5	0.264	0	0.389	0.768
G1X4S9	G1XLV4	756982.G1 X4S9	756982.G1 XLV4	0	0	0	0	0.1	0.36	0	0.306	0.565
G1X4S9	G1XLV9	756982.G1 X4S9	756982.G1 XLV9	0	0	0.373	0	0.25 1	0	0	0.356	0.671
G1X4S9	G1XT67	756982.G1 X4S9	756982.G1 XT67	0.06	0	0	0	0	0.229	0	0.245	0.405
G1X4X7	MCM7	756982.G1 X4X7	756982.G1 X7C5	0	0	0	0	0.61 8	0.978	0.946	0.949	0.999
G1X4X7	G1XE09	756982.G1 X4X7	756982.G1 XE09	0	0	0	0	0.38 9	0.175	0	0.639	0.802
G1X4X7	G1XFS2	756982.G1 X4X7	756982.G1 XFS2	0	0	0	0	0.41 2	0.472	0	0.257	0.749
G1X4X7	G1XGS2	756982.G1 X4X7	756982.G1 XGS2	0	0	0	0	0.58	0.512	0	0.675	0.928
G1X4X7	G1XLV4	756982.G1 X4X7	756982.G1 XLV4	0	0	0	0	0.10 1	0.228	0.702	0	0.775
G1X4X7	G1XMH7	756982.G1 X4X7	756982.G1 XMH7	0	0	0	0	0.19 9	0	0	0.316	0.429
G1X4X7	G1XT19	756982.G1 X4X7	756982.G1 XT19	0	0	0	0	0.37 2	0.504	0.87	0.598	0.982
G1X591	G1X7A4	756982.G1 X591	756982.G1 X7A4	0	0	0	0	0	0.495	0	0	0.495
G1X591	G1XJZ4	756982.G1 X591	756982.G1 XJZ4	0	0	0	0	0	0	0	0.619	0.619
G1X591	G1XKY4	756982.G1 X591	756982.G1 XKY4	0	0	0	0	0	0.494	0	0.32	0.641
G1X591	G1XTY2	756982.G1 X591	756982.G1 XTY2	0	0	0	0	0	0	0	0.621	0.621
G1X591	G1XV10	756982.G1 X591	756982.G1 XV10	0	0	0	0	0	0	0	0.42	0.42
G1X5B3	G1XAK8	756982.G1 X5B3	756982.G1 XAK8	0	0	0	0	0.14 2	0	0.6	0.353	0.759
G1X5B3	G1XED5	756982.G1	756982.G1	0	0	0	0	0	0	0.869	0.425	0.921

		X5B3	XED5									
G1X5B3	G1XG07	756982.G1 X5B3	756982.G1 XG07	0	0	0	0	0.41 7	0	0	0	0.417
G1X5B3	G1XHC4	756982.G1 X5B3	756982.G1 XHC4	0	0	0	0	0.31 1	0	0	0.468	0.618
G1X5B3	G1XV10	756982.G1 X5B3	756982.G1 XV10	0	0	0	0	0	0.428	0	0	0.428
G1X5K1	G1X7G3	756982.G1 X5K1	756982.G1 X7G3	0	0	0	0	0	0	0	0.408	0.408
G1X5K1	G1XG07	756982.G1 X5K1	756982.G1 XG07	0	0	0	0	0.31 8	0.496	0	0.447	0.793
G1X5K1	G1XJZ4	756982.G1 X5K1	756982.G1 XJZ4	0	0	0	0	0.33 1	0.194	0	0	0.438
G1X5K1	G1XLU8	756982.G1 X5K1	756982.G1 XLU8	0	0	0	0	0	0.648	0	0.173	0.696
G1X5K1	G1XLV4	756982.G1 X5K1	756982.G1 XLV4	0	0	0	0	0	0.363	0	0.252	0.503
G1X5K1	G1XTY2	756982.G1 X5K1	756982.G1 XTY2	0	0	0	0	0	0.669	0	0.172	0.714
G1X5K1	G1XUH5	756982.G1 X5K1	756982.G1 XUH5	0	0	0	0	0.12 4	0	0.599	0.166	0.681
G1X5K5	G1XNK0	756982.G1 X5K5	756982.G1 XNK0	0	0	0	0	0.12 7	0	0.993	0.348	0.996
G1X5K5	G1XNK1	756982.G1 X5K5	756982.G1 XNK1	0	0	0	0	0	0	0.993	0.348	0.995
G1X5K5	G1XV10	756982.G1 X5K5	756982.G1 XV10	0	0	0	0	0	0	0.398	0.097	0.433
G1X5Y6	G1XJL2	756982.G1 X5Y6	756982.G1 XJL2	0	0	0	0	0	0.51	0	0.397	0.692
G1X618	G1X6W7	756982.G1 X618	756982.G1 X6W7	0	0	0	0	0.39 4	0	0	0.21	0.501
G1X618	G1XEP6	756982.G1 X618	756982.G1 XEP6	0.056	0	0	0	0.65	0	0	0.524	0.829
G1X618	G1XLV9	756982.G1 X618	756982.G1 XLV9	0	0	0	0	0.39	0	0	0.286	0.546
G1X645	G1X7A4	756982.G1 X645	756982.G1 X7A4	0	0	0	0	0.13 9	0	0	0.393	0.455
G1X645	G1X7V7	756982.G1 X645	756982.G1 X7V7	0	0	0	0	0	0	0	0.425	0.425
G1X6C3	MCM7	756982.G1 X6C3	756982.G1 X7C5	0	0	0	0	0.18 8	0	0.744	0	0.783
G1X6C3	G1XE09	756982.G1	756982.G1	0	0	0	0	0	0.518	0	0.259	0.628

		X6C3	XE09									
G1X6C3	G1XER5	756982.G1 X6C3	756982.G1 XER5	0	0	0	0	0	0	0.744	0.26	0.802
G1X6C3	G1XFS2	756982.G1 X6C3	756982.G1 XFS2	0	0	0	0	0	0	0.774	0	0.774
G1X6C3	G1XGS2	756982.G1 X6C3	756982.G1 XGS2	0	0	0	0	0	0	0.744	0	0.744
G1X6C3	G1XT19	756982.G1 X6C3	756982.G1 XT19	0	0	0	0	0	0	0.744	0	0.744
G1X6W7	G1XBE9	756982.G1 X6W7	756982.G1 XBE9	0	0	0	0	0.641	0	0	0	0.641
G1X6W7	G1XCI8	756982.G1 X6W7	756982.G1 XCI8	0	0	0	0	0	0	0	0.447	0.447
G1X6W7	G1XEP6	756982.G1 X6W7	756982.G1 XEP6	0	0	0	0	0.349	0	0	0.174	0.439
G1X6W7	G1XEY1	756982.G1 X6W7	756982.G1 XEY1	0	0	0	0	0	0	0.824	0	0.824
G1X6W7	G1XHC4	756982.G1 X6W7	756982.G1 XHC4	0	0	0	0	0.143	0.517	0	0.117	0.603
G1X6W7	G1XIG5	756982.G1 X6W7	756982.G1 XIG5	0	0	0	0	0.392	0.665	0	0	0.788
G1X6W7	G1XQ67	756982.G1 X6W7	756982.G1 XQ67	0	0	0	0	0	0	0.824	0	0.824
G1X6W7	G1XUH5	756982.G1 X6W7	756982.G1 XUH5	0.042	0	0	0	0.326	0	0	0.525	0.667
G1X6Y5	G1XCX4	756982.G1 X6Y5	756982.G1 XCX4	0	0	0	0	0	0	0	0.443	0.443
G1X6Y5	G1XRF2	756982.G1 X6Y5	756982.G1 XRF2	0	0	0	0	0	0	0	0.442	0.442
G1X6Y5	G1XT84	756982.G1 X6Y5	756982.G1 XT84	0	0	0	0	0	0	0.993	0	0.993
G1X7A4	G1X7V7	756982.G1 X7A4	756982.G1 X7V7	0	0	0	0	0	0	0	0.494	0.494
G1X7A4	G1XJZ4	756982.G1 X7A4	756982.G1 XJZ4	0	0	0	0	0	0.629	0	0.448	0.786
G1X7A4	G1XKX4	756982.G1 X7A4	756982.G1 XKX4	0	0	0	0	0.569	0.156	0	0.422	0.771
G1X7A4	G1XNK0	756982.G1 X7A4	756982.G1 XNK0	0.064	0	0	0	0.333	0	0	0.273	0.506
G1X7A4	G1XQ38	756982.G1 X7A4	756982.G1 XQ38	0.061	0	0	0	0	0	0.449	0.166	0.531
G1X7A4	G1XT67	756982.G1	756982.G1	0.076	0	0	0	0.22	0.19	0	0.168	0.451

		X7A4	XT67					2				
G1X7A4	G1XV10	756982.G1X7A4	756982.G1XV10	0	0	0	0	0.347	0	0	0.323	0.539
G1X7A4	G1XV29	756982.G1X7A4	756982.G1XV29	0	0	0	0	0.288	0.526	0	0.423	0.788
G1X7E7	G1XBB2	756982.G1X7E7	756982.G1XBB2	0	0	0	0	0.335	0	0	0.263	0.489
G1X7E7	G1XH20	756982.G1X7E7	756982.G1XH20	0	0	0	0	0	0	0.715	0	0.715
G1X7E7	G1XLV9	756982.G1X7E7	756982.G1XLV9	0	0	0	0	0.589	0	0	0.269	0.687
G1X7E7	G1XQ38	756982.G1X7E7	756982.G1XQ38	0	0	0	0	0	0	0.718	0	0.718
G1X7E7	G1XR79	756982.G1X7E7	756982.G1XR79	0	0	0	0	0.142	0	0	0.337	0.407
G1X7E7	G1XTC2	756982.G1X7E7	756982.G1XTC2	0	0	0	0	0.549	0	0	0	0.549
G1X7G3	MCM7	756982.G1X7G3	756982.G1X7C5	0	0	0.323	0	0.136	0	0	0.101	0.428
G1X7G3	G1XB71	756982.G1X7G3	756982.G1XB71	0	0	0	0	0.349	0	0	0.132	0.411
G1X7G3	G1XBE9	756982.G1X7G3	756982.G1XBE9	0	0	0	0	0.7	0.987	0.945	0.646	0.999
G1X7G3	G1XIG5	756982.G1X7G3	756982.G1XIG5	0	0	0	0	0.097	0	0.599	0	0.622
G1X7G3	G1XJZ4	756982.G1X7G3	756982.G1XJZ4	0	0	0	0	0	0	0.475	0	0.475
G1X7G3	G1XKY4	756982.G1X7G3	756982.G1XKY4	0	0	0	0	0	0	0.475	0	0.475
G1X7V0	G1XCI8	756982.G1X7V0	756982.G1XCI8	0	0	0	0	0.239	0	0	0.545	0.639
G1X7V7	G1XI36	756982.G1X7V7	756982.G1XI36	0	0	0	0	0	0	0	0.42	0.42
G1X8L2	G1XCX4	756982.G1X8L2	756982.G1XCX4	0	0	0	0	0	0	0	0.421	0.421
G1X8L2	G1XUH5	756982.G1X8L2	756982.G1XUH5	0	0	0	0	0	0	0.455	0	0.455
G1X8N2	G1XAJ0	756982.G1X8N2	756982.G1XAJ0	0	0	0	0	0.347	0	0	0.78	0.85
G1X8N2	G1XCI8	756982.G1X8N2	756982.G1XCI8	0	0	0	0	0.096	0	0	0.415	0.449
G1X8N2	G1XG07	756982.G1	756982.G1	0	0	0	0	0.20	0	0	0.468	0.557

		X8N2	XG07					1				
G1X8N2	G1XLK0	756982.G1X8N2	756982.G1XLK0	0	0	0	0	0	0	0	0.406	0.406
G1X8N3	G1XAK8	756982.G1X8N3	756982.G1XAK8	0	0	0	0	0.143	0.534	0.598	0.875	0.977
G1X8N3	G1XG07	756982.G1X8N3	756982.G1XG07	0	0	0	0	0	0.505	0	0.174	0.574
G1X8N3	G1XHC4	756982.G1X8N3	756982.G1XHC4	0	0	0	0	0.346	0	0	0.173	0.436
G1X8N3	G1XIG5	756982.G1X8N3	756982.G1XIG5	0	0	0	0	0.142	0.345	0.601	0.268	0.814
G1X8N3	G1XTC2	756982.G1X8N3	756982.G1XTC2	0	0	0	0	0.262	0	0	0.233	0.41
G1X8N6	G1XIG5	756982.G1X8N6	756982.G1XIG5	0	0	0	0	0	0.495	0	0.269	0.615
G1X9L3	G1XLV4	756982.G1X9L3	756982.G1XLV4	0	0	0	0	0.192	0.5	0	0.406	0.739
G1XAJ0	G1XFG1	756982.G1XAJ0	756982.G1XFG1	0	0	0	0	0.075	0	0.856	0	0.861
G1XAJ0	G1XFQ1	756982.G1XAJ0	756982.G1XFQ1	0	0	0	0	0	0.212	0	0.448	0.546
G1XAJ0	G1XI99	756982.G1XAJ0	756982.G1XI99	0	0	0	0	0.075	0	0.993	0	0.993
G1XAJ0	G1XIF7	756982.G1XAJ0	756982.G1XIF7	0	0	0	0	0	0.473	0	0	0.473
G1XAJ0	G1XJL2	756982.G1XAJ0	756982.G1XJL2	0	0	0	0	0.114	0.16	0	0.259	0.4
G1XAJ0	G1XKT9	756982.G1XAJ0	756982.G1XKT9	0	0	0	0	0.203	0.162	0	0.394	0.56
G1XAJ0	G1XLK2	756982.G1XAJ0	756982.G1XLK2	0	0	0	0	0	0.609	0	0.473	0.785
G1XAJ0	G1XNK0	756982.G1XAJ0	756982.G1XNK0	0	0	0	0	0.355	0	0	0.133	0.417
G1XAJ0	G1XSV0	756982.G1XAJ0	756982.G1XSV0	0	0	0	0	0.114	0	0	0.551	0.585
G1XAJ0	G1XTA1	756982.G1XAJ0	756982.G1XTA1	0	0	0	0	0	0	0.993	0.135	0.994
G1XAJ0	G1XV29	756982.G1XAJ0	756982.G1XV29	0	0	0	0	0.199	0.229	0	0.635	0.755
G1XAK8	G1XB71	756982.G1XAK8	756982.G1XB71	0	0	0	0	0	0.269	0	0.236	0.418
G1XAK8	G1XHC4	756982.G1	756982.G1	0	0	0	0	0.13	0	0	0.47	0.522

		XAK8	XHC4					5				
G1XAK8	G1XR79	756982.G1 XAK8	756982.G1 XR79	0	0	0	0	0.13 5	0	0	0.339	0.404
G1XAL6	MCM7	756982.G1 XAL6	756982.G1 X7C5	0	0	0	0	0.14 1	0.557	0	0	0.603
G1XB71	G1XCI8	756982.G1 XB71	756982.G1 XCI8	0	0	0	0	0.20 2	0.62	0	0	0.684
G1XBB2	G1XD36	756982.G1 XBB2	756982.G1 XD36	0.06	0	0	0	0	0	0	0.617	0.625
G1XBB2	G1XJZ4	756982.G1 XBB2	756982.G1 XJZ4	0	0	0	0	0.23 4	0	0	0.611	0.689
G1XBB2	G1XKT9	756982.G1 XBB2	756982.G1 XKT9	0.06	0	0	0	0.35 4	0	0	0.413	0.612
G1XBB2	G1XNK0	756982.G1 XBB2	756982.G1 XNK0	0.056	0	0	0	0	0	0.356	0.259	0.51
G1XBB2	G1XNK1	756982.G1 XBB2	756982.G1 XNK1	0.056	0	0	0	0	0	0.356	0.259	0.51
G1XBB2	G1XTD6	756982.G1 XBB2	756982.G1 XTD6	0.062	0	0	0	0.32 7	0.226	0.366	0.155	0.691
G1XBE9	G1XE09	756982.G1 XBE9	756982.G1 XE09	0	0	0	0	0	0	0	0.4	0.4
G1XBE9	G1XHC4	756982.G1 XBE9	756982.G1 XHC4	0	0	0	0	0.21 3	0.263	0	0.235	0.518
G1XBE9	G1XIG5	756982.G1 XBE9	756982.G1 XIG5	0	0	0	0	0.28 8	0	0.599	0	0.702
G1XBE9	G1XJZ4	756982.G1 XBE9	756982.G1 XJZ4	0	0	0	0	0.23 9	0	0.475	0.236	0.668
G1XBE9	G1XKY4	756982.G1 XBE9	756982.G1 XKY4	0	0	0	0	0	0	0.475	0	0.475
G1XBE9	G1XTY2	756982.G1 XBE9	756982.G1 XTY2	0	0	0	0	0	0.512	0	0.242	0.614
G1XCB0	G1XLK2	756982.G1 XCB0	756982.G1 XLK2	0	0	0	0	0.09 3	0	0	0.687	0.704
G1XCB0	G1XRV7	756982.G1 XCB0	756982.G1 XRV7	0	0	0	0	0	0	0.912	0	0.912
G1XCI8	G1XLK2	756982.G1 XCI8	756982.G1 XLK2	0	0	0	0	0	0	0.426	0	0.426
G1XCX4	G1XIF7	756982.G1 XCX4	756982.G1 XIF7	0	0	0	0	0	0	0	0.423	0.423
G1XCX4	G1XIG5	756982.G1 XCX4	756982.G1 XIG5	0	0	0	0	0.23 5	0	0	0.263	0.412
G1XCX4	G1XKY4	756982.G1	756982.G1	0	0	0	0	0.19	0.615	0.993	0.454	0.999

		XCX4	XKY4									
G1XCX4	G1XLU8	756982.G1 XCX4	756982.G1 XLU8	0	0	0	0	0	0	0.993	0.294	0.995
G1XCX4	G1XR79	756982.G1 XCX4	756982.G1 XR79	0	0	0	0	0	0	0.794	0	0.794
G1XCX4	G1XRF2	756982.G1 XCX4	756982.G1 XRF2	0	0	0	0	0.354	0	0	0.408	0.601
G1XCX4	G1XT84	756982.G1 XCX4	756982.G1 XT84	0	0	0	0	0	0	0	0.443	0.443
G1XD36	G1XG07	756982.G1 XD36	756982.G1 XG07	0	0	0	0	0	0.218	0	0.354	0.473
G1XD36	G1XKT9	756982.G1 XD36	756982.G1 XKT9	0.044	0	0	0	0.308	0	0	0.721	0.799
G1XE09	MCM7	756982.G1 XE09	756982.G1 X7C5	0	0	0	0	0.406	0.635	0.599	0.854	0.986
G1XE09	G1XER5	756982.G1 XE09	756982.G1 XER5	0	0	0	0	0	0.176	0	0.648	0.698
G1XE09	G1XFS2	756982.G1 XE09	756982.G1 XFS2	0	0	0	0	0.402	0	0.606	0.538	0.882
G1XE09	G1XGS2	756982.G1 XE09	756982.G1 XGS2	0	0	0	0	0.197	0.518	0	0.303	0.707
G1XE09	G1XHC4	756982.G1 XE09	756982.G1 XHC4	0	0	0	0	0.183	0	0	0.356	0.451
G1XE09	G1XIG5	756982.G1 XE09	756982.G1 XIG5	0	0	0	0	0	0.217	0.597	0.266	0.748
G1XE09	G1XJZ4	756982.G1 XE09	756982.G1 XJZ4	0	0	0	0.369	0	0.196	0	0.46	0.418
G1XE09	G1XLV9	756982.G1 XE09	756982.G1 XLV9	0	0	0	0	0.135	0.509	0	0.215	0.637
G1XE09	G1XMH7	756982.G1 XE09	756982.G1 XMH7	0	0	0	0	0.267	0	0	0.57	0.671
G1XE09	G1XT19	756982.G1 XE09	756982.G1 XT19	0	0	0	0	0.186	0	0.601	0.4	0.788
G1XER5	G1XFS2	756982.G1 XER5	756982.G1 XFS2	0	0	0	0	0.11	0	0.744	0.123	0.783
G1XER5	G1XGS2	756982.G1 XER5	756982.G1 XGS2	0	0	0	0	0.222	0	0	0.503	0.597
G1XER5	G1XMH7	756982.G1 XER5	756982.G1 XMH7	0	0	0	0	0.188	0.505	0	0.388	0.733
G1XEY1	G1XI36	756982.G1 XEY1	756982.G1 XI36	0	0	0	0	0	0	0	0.549	0.549
G1XEY1	G1XNU1	756982.G1	756982.G1	0	0	0	0	0	0	0.401	0	0.401

		XEY1	XNU1									
G1XEY1	G1XQ38	756982.G1XEY1	756982.G1XQ38	0	0	0	0.113	0.354	0	0	0.642	0.713
G1XEY1	G1XQ67	756982.G1XEY1	756982.G1XQ67	0	0	0	0.841	0.548	0.512	0.824	0.909	0.964
G1XFG1	G1XI36	756982.G1XFG1	756982.G1XI36	0	0	0	0	0	0	0.975	0	0.975
G1XFG1	G1XLK2	756982.G1XFG1	756982.G1XLK2	0	0	0	0	0	0	0.456	0	0.456
G1XFG1	G1XTA1	756982.G1XFG1	756982.G1XTA1	0	0	0	0	0	0	0.856	0	0.856
G1XFQ1	G1XNH2	756982.G1XFQ1	756982.G1XNH2	0	0	0	0	0	0	0	0.754	0.754
G1XFS2	MCM7	756982.G1XFS2	756982.G1X7C5	0	0	0	0	0.818	0	0.599	0.492	0.96
G1XFS2	G1XG07	756982.G1XFS2	756982.G1XG07	0	0	0	0	0.572	0	0	0	0.572
G1XFS2	G1XGS2	756982.G1XFS2	756982.G1XGS2	0	0	0	0	0.613	0.513	0.601	0.491	0.957
G1XFS2	G1XMH7	756982.G1XFS2	756982.G1XMH7	0	0	0	0	0.143	0	0	0.338	0.408
G1XFS2	G1XT19	756982.G1XFS2	756982.G1XT19	0	0	0	0	0.195	0.512	0	0.717	0.879
G1XG07	MCM7	756982.G1XG07	756982.G1X7C5	0	0	0	0	0.396	0	0	0.113	0.441
G1XG07	G1XHC4	756982.G1XG07	756982.G1XHC4	0	0	0	0	0.142	0.263	0	0.147	0.414
G1XG07	G1XIG5	756982.G1XG07	756982.G1XIG5	0	0	0	0	0.142	0.42	0	0.1	0.513
G1XG07	G1XLV4	756982.G1XG07	756982.G1XLV4	0	0	0	0	0	0.356	0	0.395	0.594
G1XGS2	MCM7	756982.G1XGS2	756982.G1X7C5	0	0	0	0	0.792	0.221	0.972	0.558	0.998
G1XGS2	G1XT19	756982.G1XGS2	756982.G1XT19	0	0	0	0	0.188	0.229	0.933	0.442	0.973
G1XH20	G1XQ38	756982.G1XH20	756982.G1XQ38	0	0	0	0	0	0	0.718	0	0.718
G1XHC4	MCM7	756982.G1XHC4	756982.G1X7C5	0	0	0	0	0.39	0	0	0.139	0.452
G1XHC4	G1XJZ4	756982.G1XHC4	756982.G1XJZ4	0	0	0	0	0.193	0.571	0	0.097	0.66
G1XHC4	G1XLV4	756982.G1	756982.G1	0	0	0	0	0.08	0.475	0	0.097	0.528

		XHC4	XLV4					5				
G1XHC4	G1XLV9	756982.G1XHC4	756982.G1XLV9	0	0	0	0	0.106	0.571	0	0	0.6
G1XHC4	G1XNK0	756982.G1XHC4	756982.G1XNK0	0	0	0	0	0.138	0.509	0	0.15	0.609
G1XHC4	G1XT19	756982.G1XHC4	756982.G1XT19	0	0	0	0	0.143	0	0	0.332	0.403
G1XHC4	G1XTC2	756982.G1XHC4	756982.G1XTC2	0	0	0	0	0.199	0.151	0	0.771	0.831
G1XI36	G1XI99	756982.G1XI36	756982.G1XI99	0	0	0	0	0	0	0.993	0	0.993
G1XI99	G1XLK2	756982.G1XI99	756982.G1XLK2	0	0	0	0	0	0	0.456	0	0.456
G1XI99	G1XTA1	756982.G1XI99	756982.G1XTA1	0	0	0	0	0	0	0.985	0	0.985
G1XIG5	G1XJZ4	756982.G1XIG5	756982.G1XJZ4	0	0	0	0	0.195	0.597	0.491	0.12	0.835
G1XIG5	G1XKY4	756982.G1XIG5	756982.G1XKY4	0	0	0	0	0	0.162	0.804	0.261	0.868
G1XIV2	G1XJZ4	756982.G1XIV2	756982.G1XJZ4	0	0	0	0	0	0	0.713	0.234	0.771
G1XIV2	G1XKX3	756982.G1XIV2	756982.G1XKX3	0	0	0	0	0	0	0.718	0	0.718
G1XIV2	G1XKY4	756982.G1XIV2	756982.G1XKY4	0	0	0	0	0	0	0.711	0	0.711
G1XJL2	G1XJZ4	756982.G1XJL2	756982.G1XJZ4	0	0	0	0	0	0.211	0	0.278	0.406
G1XJL2	G1XMG3	756982.G1XJL2	756982.G1XMG3	0	0	0	0	0.497	0.425	0	0.41	0.814
G1XJZ4	G1XKY4	756982.G1XJZ4	756982.G1XKY4	0	0	0	0.856	0	0.669	0.774	0.868	0.932
G1XJZ4	G1XLU8	756982.G1XJZ4	756982.G1XLU8	0	0	0	0	0	0.588	0.473	0.472	0.875
G1XKH1	G1XTD6	756982.G1XKH1	756982.G1XTD6	0	0	0	0	0.411	0.664	0	0.163	0.82
G1XKT9	G1XNK0	756982.G1XKT9	756982.G1XNK0	0.058	0	0	0	0.404	0	0	0.138	0.474
G1XKT9	G1XT67	756982.G1XKT9	756982.G1XT67	0.047	0	0	0	0.198	0	0	0.284	0.405
G1XKX3	G1XNU1	756982.G1XKX3	756982.G1XNU1	0	0	0	0	0	0	0.993	0	0.993
G1XKX4	G1XMX9	756982.G1	756982.G1	0.05	0	0	0	0	0	0.278	0.26	0.448

		XKX4	XMX9									
G1XKX4	G1XN30	756982.G1XKX4	756982.G1XN30	0.049	0	0	0	0	0	0.77	0.099	0.786
G1XKX4	G1XV10	756982.G1XKX4	756982.G1XV10	0	0	0	0	0.355	0.224	0	0.256	0.595
G1XKY4	G1XLU8	756982.G1XKY4	756982.G1XLU8	0	0	0	0	0.525	0.639	0.473	0.461	0.945
G1XKY4	G1XNK0	756982.G1XKY4	756982.G1XNK0	0	0	0	0	0	0	0.677	0.097	0.696
G1XLK2	G1XSV0	756982.G1XLK2	756982.G1XSV0	0	0	0	0	0.199	0.862	0.993	0.96	0.999
G1XLK2	G1XTA1	756982.G1XLK2	756982.G1XTA1	0	0	0	0	0	0	0.491	0.101	0.523
G1XLK2	G1XV29	756982.G1XLK2	756982.G1XV29	0	0	0	0	0	0	0	0.767	0.767
G1XLU8	MCM7	756982.G1XLU8	756982.G1X7C5	0	0	0	0	0	0.512	0	0.746	0.871
G1XLU8	G1XLV4	756982.G1XLU8	756982.G1XLV4	0	0	0	0	0	0.459	0	0.172	0.533
G1XLU8	G1XTY2	756982.G1XLU8	756982.G1XTY2	0	0	0	0	0	0	0	0.419	0.419
G1XLV4	MCM7	756982.G1XLV4	756982.G1X7C5	0	0	0	0	0.076	0.272	0.702	0.3	0.841
G1XLV4	G1XT19	756982.G1XLV4	756982.G1XT19	0	0	0	0	0	0.371	0	0.312	0.549
G1XLV4	G1XTC2	756982.G1XLV4	756982.G1XTC2	0	0	0	0	0.075	0.538	0	0.331	0.689
G1XLV9	G1XRF2	756982.G1XLV9	756982.G1XRF2	0	0	0	0	0	0.525	0	0	0.525
G1XLV9	G1XTC2	756982.G1XLV9	756982.G1XTC2	0	0	0	0	0.828	0	0	0	0.828
G1XMX9	G1XN30	756982.G1XMX9	756982.G1XN30	0.044	0	0	0	0	0	0.667	0.21	0.727
G1XNH2	G1XT67	756982.G1XNH2	756982.G1XT67	0	0	0	0	0	0	0	0.428	0.428
G1XNK0	G1XNK1	756982.G1XNK0	756982.G1XNK1	0	0	0.476	0	0.565	0	0.993	0.713	0.999
G1XNK0	G1XT67	756982.G1XNK0	756982.G1XT67	0.054	0	0	0	0.112	0	0.993	0.266	0.995
G1XNK1	G1XT67	756982.G1XNK1	756982.G1XT67	0.054	0	0	0	0.075	0	0.993	0	0.993
G1XNU1	G1XQ38	756982.G1	756982.G1	0.046	0	0	0	0	0	0.401	0	0.404

		XNU1	XQ38									
G1XQ38	G1XQ67	756982.G1XQ38	756982.G1XQ67	0	0	0	0	0.338	0	0	0.458	0.626
G1XRF2	G1XT84	756982.G1XRF2	756982.G1XT84	0	0	0	0	0	0	0	0.442	0.442
G1XSV0	G1XV29	756982.G1XSV0	756982.G1XV29	0	0	0	0	0.105	0	0	0.759	0.775
G1XT19	MCM7	756982.G1XT19	756982.G1X7C5	0	0	0	0	0.579	0.872	0.993	0.856	0.999
G1XT67	G1XV10	756982.G1XT67	756982.G1XV10	0	0	0	0	0.2	0.495	0	0.456	0.761
G1XTC2	G1XUH5	756982.G1XTC2	756982.G1XUH5	0	0	0	0	0	0.512	0	0.4	0.695
G1XV29	MCM7	756982.G1XV29	756982.G1X7C5	0	0	0	0	0	0	0	0.412	0.412

Table S4 Reads and base filter information statistics for DAP-Seq.

Sample	Clean_Reads_Num	HQ_Clean_Reads_Num(%)	Read_length	adapter(%)	low_quality (%)	polyA (%)	N(%)
IP*	82230778	82006654(99.73%)	150 + 150	81210(0.1%)	285204(0.17%)	0(0.0%)	624 (0.0%)
input	78182218	77980050(99.74%)	150 + 150	55538(0.07%)	293180(0.19%)	0(0.0%)	80 (0.0%)

Sample	Clean_Data (bp)	Q20 (%)	Q30 (%)	N(%)	GC (%)	HQ_Clean_Data(bp)	HQ_Q20 (%)	HQ_Q30 (%)	HQ_N (%)	HQ_GC (%)
IP	12334616700	12108684146 (98.17%)	11685889178 (94.74%)	182029 (0.0%)	5314321787 (43.08%)	11643736417	11452448708 (98.36%)	11070092028 (95.07%)	166042 (0.0%)	4950787686 (42.52%)
Input	11727332700	11409434184 (97.29%)	10888301186 (92.85%)	35600 (0.0%)	5204902863 (44.38%)	11149500511	10867813275 (97.47%)	10386963540 (93.16%)	33357 (0.0%)	4904224535 (43.98%)

*IP indicates the experimental group; Input indicates the negative control in DAP-Seq analysis.

Table S5 Annotation of metabolites of the Δ*Aoste12* mutant vs. WT strains.
